# Opsonization Inveigles Macrophages Engulfing Carrier‐Free Bilirubin/JPH203 Nanoparticles to Suppress Inflammation for Osteoarthritis Therapy

**DOI:** 10.1002/advs.202400713

**Published:** 2024-04-09

**Authors:** Huirong Huang, Shimin Zheng, Jianing Wu, Xindan Liang, Shengjie Li, Pengfei Mao, Zhinan He, Yahui Chen, Lining Sun, Xinyu Zhao, Aimin Cai, Luhui Wang, Huixiang Sheng, Qing Yao, Ruijie Chen, Ying‐Zheng Zhao, Longfa Kou

**Affiliations:** ^1^ Wenzhou Municipal Key Laboratory of Pediatric Pharmacy, Department of Pharmacy The Second Affiliated Hospital and Yuying Children's Hospital of Wenzhou Medical University Wenzhou 325027 China; ^2^ Key Laboratory of Structural Malformations in Children of Zhejiang Province Wenzhou 325027 China; ^3^ School of Pharmaceutical Sciences Wenzhou Medical University Wenzhou 325035 China; ^4^ Department of Ultrasonography The First Affiliated Hospital of Wenzhou Medical University Wenzhou 325015 China

**Keywords:** bilirubin, JPH203, macrophage, opsonization, osteoarthritis

## Abstract

Osteoarthritis (OA) is a chronic inflammatory disease characterized by cartilage destruction, synovitis, and osteophyte formation. Disease‐modifying treatments for OA are currently lacking. Because inflammation mediated by an imbalance of M1/M2 macrophages in the synovial cavities contributes to OA progression, regulating the M1 to M2 polarization of macrophages can be a potential therapeutic strategy. Basing on the inherent immune mechanism and pathological environment of OA, an immunoglobulin G‐conjugated bilirubin/JPH203 self‐assembled nanoparticle (IgG/BRJ) is developed, and its therapeutic potential for OA is evaluated. After intra‐articular administration, IgG conjugation facilitates the recognition and engulfment of nanoparticles by the M1 macrophages. The internalized nanoparticles disassemble in response to the increased oxidative stress, and the released bilirubin (BR) and JPH203 scavenge reactive oxygen species (ROS), inhibit the nuclear factor kappa‐B pathway, and suppress the activated mammalian target of rapamycin pathway, result in the repolarization of macrophages and enhance M2/M1 ratios. Suppression of the inflammatory environment by IgG/BRJ promotes cartilage protection and repair in an OA rat model, thereby improving therapeutic outcomes. This strategy of opsonization involving M1 macrophages to engulf carrier‐free BR/JPH203 nanoparticles to suppress inflammation for OA therapy holds great potential for OA intervention and treatment.

## Graphical abstract

An IgG‐opsonized carrier‐free bilirubin/JPH203 nanoparticle has been successfully developed. This nanoparticle exhibits precise targeting capabilities and modulates macrophage M1‐M2 polarization, resulting in the effective mitigation of inflammation and promotion of cartilage regeneration in rat models of osteoarthritis. This innovative strategy offers great promise for advancing osteoarthritis intervention and treatment in the field of nanomedicine.

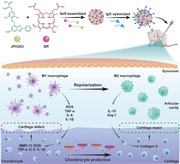

## 1 Introduction

Osteoarthritis (OA) is a chronic disease characterized by persistent inflammatory processes, degenerative transformations within articular cartilage structures, and the consequential formation of osteophytes. Symptomatic and functionally constrained OA manifests in ≈240 million individuals globally, underscoring its substantial impact on the patients' quality of life and socioeconomic strain.^[^ ^1^ ^]^ Consequently, OA is a prominent public health concern that requires expeditious attention.^[^ ^2^ ^]^ Current clinical interventions for OA include nonsteroidal anti‐inflammatory drugs (NSAIDs), glucocorticoids, and hyaluronic acid‐based adjuncts.^[^ ^3^ ^]^ Although these therapeutic modalities are efficient in mitigating pain and inflammatory responses, they are ineffective in fundamentally impeding OA progression.^[^ ^4^ ^]^ Moreover, prolonged use of NSAIDs and glucocorticoids raises concerns regarding the emergence of deleterious effects,^[^ ^5^ ^]^ emphasizing the need for additional research to develop effective therapeutic strategies.

Macrophages are intrinsic immune cells that play a pivotal role in the human physiological environment and are mainly involved in the initiation and progression of diverse inflammatory pathologies.^[^ ^6^ ^]^ Notably, macrophages are extensively involved in the OA trajectory.^[^ ^7^ ^]^ Macrophages adopt distinct subtypes throughout the various phases of inflammation. The response to diverse pathogenic stimuli or maladaptive microenvironments prompts phenotypic divergence toward M1 or M2 phenotypes, capable of performing certain specific functions.^[^ ^8^ ^]^ M1 macrophages release substantial pro‐inflammatory cytokines such as interleukin (IL)‐1, IL‐6, tumor necrosis factor‐α (TNF‐α), and reactive oxygen species (ROS), which leads to the augmentation of inflammatory gradients conducive to antimicrobial or antitumor.^[^ ^9^ ^]^ Conversely, M2 macrophages secrete anti‐inflammatory factors in tandem with trophic agents, thereby attenuating inflammatory responses and facilitating tissue repair.^[^ ^10^ ^]^ M1 macrophages accentuate the catabolic propensity of chondrocytes in the OA milieu, predisposing them to cartilage degenerative cascades.^[^ ^11^ ^]^ Conversely, M2 macrophages promote anabolic activities in chondrocytes and exert protective effects.^[^ ^12^ ^]^ Therefore, the orchestrated modulation of macrophage polarization, i.e., a shift from the pro‐inflammatory M1 phenotype to the anti‐inflammatory M2 phenotype, is a practical therapeutic approach for OA management.^[^ ^13^ ^]^

Understanding the characteristics of M1 macrophages is imperative for modulating the transition from M1 to M2 polarization. Elevated ROS levels constitute the cornerstone of the pathological milieu in OA, leading to the activation of M1 macrophages.^[^ ^14^ ^]^ Moreover, increased ROS concentrations activate the nuclear factor kappa‐B (NF‐κB) signaling pathway, thereby fueling M1 polarization and amplifying inflammatory cascades.^[^ ^15^ ^]^ Consequently, intervention with ROS‐scavenging antioxidants promote the M1 to M2 polarization. Xiong et al. developed biodegradable hollow‐structured manganese Prussian blue nano‐enzymes adept at ROS scavenging and efficaciously promoting M1 to M2 polarization.^[^ ^16^ ^]^ Similarly, melanin/alginate hydrogels have the potential to pivot M2 macrophages through ROS elimination.^[^ ^17^ ^]^ Bilirubin (BR), a byproduct of heme catabolism, is an endogenous antioxidant with potential implications.^[^ ^18^ ^]^ The efficacy of BR in neutralizing free radicals had been demonstrated previously, substantiating its role as a potent antioxidant.^[^ ^19^ ^]^ Furthermore, BR exhibits anti‐inflammatory, immunomodulatory, and anti‐apoptotic properties in diverse conditions, such as acute colitis, acute pancreatitis, and acute kidney injuries.^[^ ^20^ ^]^ The ability of BR to incite macrophage polarization toward the M2 phenotype has been previously reported.^[^ ^21^ ^]^ The distinctive characteristics of BR augment its potential for orchestrating the M1 to M2 transition. Moreover, the pivotal role of the mammalian target of rapamycin (mTOR) pathway in macrophage polarization has been elucidated.^[^ ^22^ ^]^ The ability of mTORC1 to augment protein and nucleotide synthesis facilitates the production of inflammatory mediators and chemokines, thereby inducing overactivation of M1 macrophages.^[^ ^23^ ^]^ The mTOR pathway inhibitors modulate macrophage polarization dynamics. Wu et al. reported that, dioscin inhibits M1 polarization and promotes M2 polarization by blocking the mTOR pathway.^[^ ^24^ ^]^ Although antioxidants and mTOR inhibitors demonstrate macrophage polarization, their individual effects are suboptimal; therefore, their synergistic effects hold great potential for accentuating M2 macrophage polarization.

Nanomedicines have garnered substantial attention because of their ability to enhance the solubility and stability of drug molecules, improve drug behavior and efficacy, and mitigate unwarranted side effects.^[^ ^25^ ^]^ Nanomedicine has diverse applications, including tumor eradication,^[^ ^26^ ^]^ inflammation control,^[^ ^27^ ^]^ and wound healing.^[^ ^28^ ^]^ Nonetheless, nanomedicines that have been designed and developed for more efficient diagnosis and disease treatment are inevitably removed by the reticuloendothelial system (RES) after intravenous injection. Nanoparticles entering the bloodstream interact with opsonin proteins, such as complement (C3, C4, and C5), immunoglobulins, C‐reactive proteins, and lectins, which are called opsonization. Subsequently, the activated M1 macrophages in the RES engulf and clear opsonized nanoparticles via highly expressed surface receptors, including Fc and complement receptors,^[^ ^30^ ^]^ resulting in poor in vivo effects of nanomedicines. Whereas, for targeting macrophages to treat OA, opsonized nanoparticles can efficiently bind to the active M1 macrophages, thereby improving the targeting precision and reducing the toxic side effects of drugs. This tailored approach facilitates the specific regulation of macrophages, transitioning them from M1 to M2 phenotype for effective OA therapy.^[^ ^31^ ^]^

**Scheme 1:**
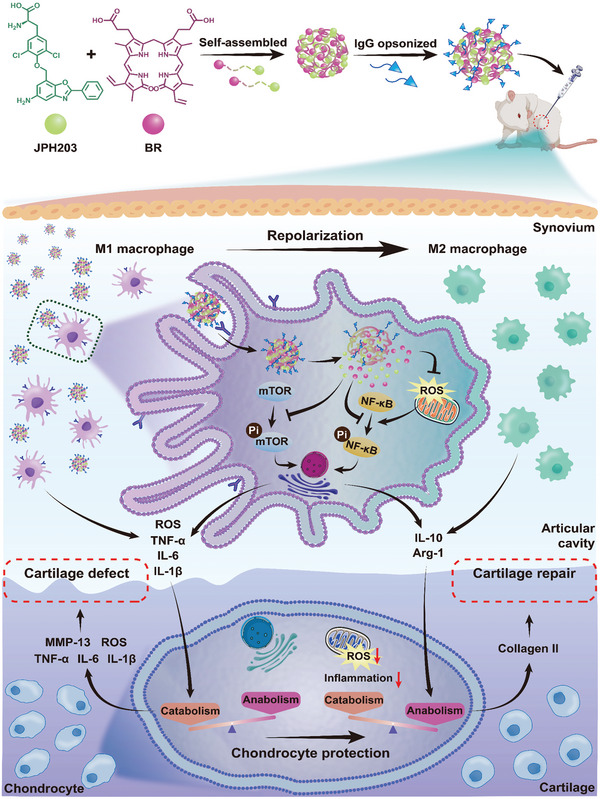
Schematic illustration of the preparation and mechanism of opsonized bilirubin/JPH203 nanoparticles (IgG/BRJ) for osteoarthritis therapy.

Based on this rationale, we developed an opsonized nanoparticle system aimed at delivering a synergistic combination of BR and JPH203 (J), a targeted mTOR inhibitor,^[^ ^32^ ^]^ to M1 macrophages. BR and JPH203 possess the inherent ability to self‐assemble and nanoparticle formation (BRJ),^[^ ^33^ ^]^ and immunoglobulin G (IgG) was used to modify BR/JPH203 nanoparticles, resulting in the formation of opsonized BR/JPH203 self‐assembled nanoparticles (IgG/BRJ) designed for M1 macrophage targeting and OA treatment (**Scheme** **1**). After intra‐articular administration, IgG conjugation enabled the nanoparticles to be recognized and engulfed by M1 macrophages. In response to the elevated oxidative stress, the internalized nanoparticles disintegrate to release BR and JPH203, which scavenge ROS, inhibit the NF‐κB pathway, and suppress the activated mTOR pathway, effectively orchestrating the transition from pro‐inflammatory M1 polarization to anti‐inflammatory M2 polarization. Suppression of the inflammatory milieu by IgG/BRJ promoted cartilage protection and repair in an OA rat model, demonstrating optimal therapeutic outcomes. Overall, the effects of IgG/BRJ highlight its potential as a promising therapeutic agent for the treatment of OA. By directly targeting the inflammatory response, protecting chondrocytes, and reshaping the chondroinflammatory microenvironment, IgG/BRJ may offer new avenues for the treatment and management of OA.

## 2 Results and Discussion

### 2.1 Preparation and Characterization of IgG/BRJ

We aimed to investigate the potential of BR, JPH203, and their combination in regulating M1‐M2 polarization and their anti‐inflammatory properties. Quantitative real‐time polymerase chain reaction (qRT‐PCR) was performed to determine the optimal concentration and ratio of BR/JPH203. BR treatment significantly downregulated the mRNA levels of pro‐inflammatory factors (iNOS, TNF‐α, and IL‐1β) (Figure S1A, Supporting Information). Particularly, low concentrations of BR (5 µm) significantly downregulated iNOS expression, whereas insignificant downregulation of IL‐1β was observed until the BR concentration reached 20 µm. Conversely, the mRNA levels of anti‐inflammatory factors (IL‐10 and Arg‐1) were upregulated by BR treatment in a concentration‐dependent manner. The mRNA levels of TNF‐α and IL‐1β exhibited remarkable downregulation at a JPH203 concentration of 20 µm (Figure S1B, Supporting Information). Similar to BR treatment, JPH203 treatment upregulated the mRNA levels of IL‐10 and Arg‐1 in a concentration‐dependent manner. Based on the outcomes of single drug tests, 20 µm of BR was administered to explore the optimal combination ratio of BR and JPH203. The mRNA levels of iNOS, TNF‐α, and IL‐1β were significantly downregulated at a BR‐to‐JPH203 combination ratio of 1:0.5 (Figure S1C, Supporting Information). Moreover, this ratio substantially enhanced the mRNA levels of IL‐10 and Arg‐1. Based on these results, a bilirubin/JPH203 nanoparticle (BRJ) ratio of 1:0.5 (20 µm and 10 µm concentrations of BR and JPH203, respectively) was adopted for subsequent experiments. The objective was to simulate the biological opsonization process by utilizing IgG to bind to the surface of BRJ nanoparticles to prepare opsonized nanoparticles (IgG/BRJ). To achieve this, the affinity of BR and JPH203 for IgG was analyzed, and molecular docking analysis was performed. The binding configurations and interactions between IgG‐BR and IgG‐JPH203 were obtained using the AutoDock Vina 1.2.2 software, and the binding energy for each interaction was generated (Figure S2, Supporting Information). The results indicated that BR and JPH203 were bound to IgG via visible hydrogen bonds. The binding energies of IgG‐BR and IgG‐JPH203 were low (‐7.613 kcal/mol and ‐8.194 kcal/mol, respectively), indicating a highly stable binding. Notably, the binding sites of BR and JPH203 with IgG are located in the Fab region, which ensures that the Fc region is exposed after adsorption onto the surface of BRJ nanoparticles, thereby facilitating targeted M1 macrophage delivery. Subsequently, the optimal IgG‐binding ratio was evaluated. The particle size, polydispersity index (PDI), and zeta potential of IgG/BRJ nanoparticles bound to different ratios of IgG (10%, 50%, and 100% of BR mass) were assessed (Figure S3A–C, Supporting Information). The results showed that the particle size gradually increased with increasing IgG ratio, ranging from 112.3 ± 2.0 nm to 141.4 ± 0.6 nm. The PDI values varied between 0.13 and 0.22, indicating acceptable uniformity. Zeta potentials remained similar across the tested ratios. Opsonized IgG/BRJ nanoparticles were developed to target activated M1 macrophages. Therefore, to optimize the binding ratio of IgG to nanoparticles, the impact of different IgG ratios on the M1 cellular uptake of nanoparticles were assessed. The cellular uptake of nanoparticles bound to 10%, 20%, and 40% IgG increased to some extent with extended incubation time (Figure S3D,E, Supporting Information). Among these, 20% IgG/BRJ nanoparticles exhibited maximum ingestion after 4 h of incubation; therefore, an IgG‐binding ratio of 20% was selected for the preparation of IgG/BRJ nanoparticles and subsequent biological evaluation.

**Figure 1:**
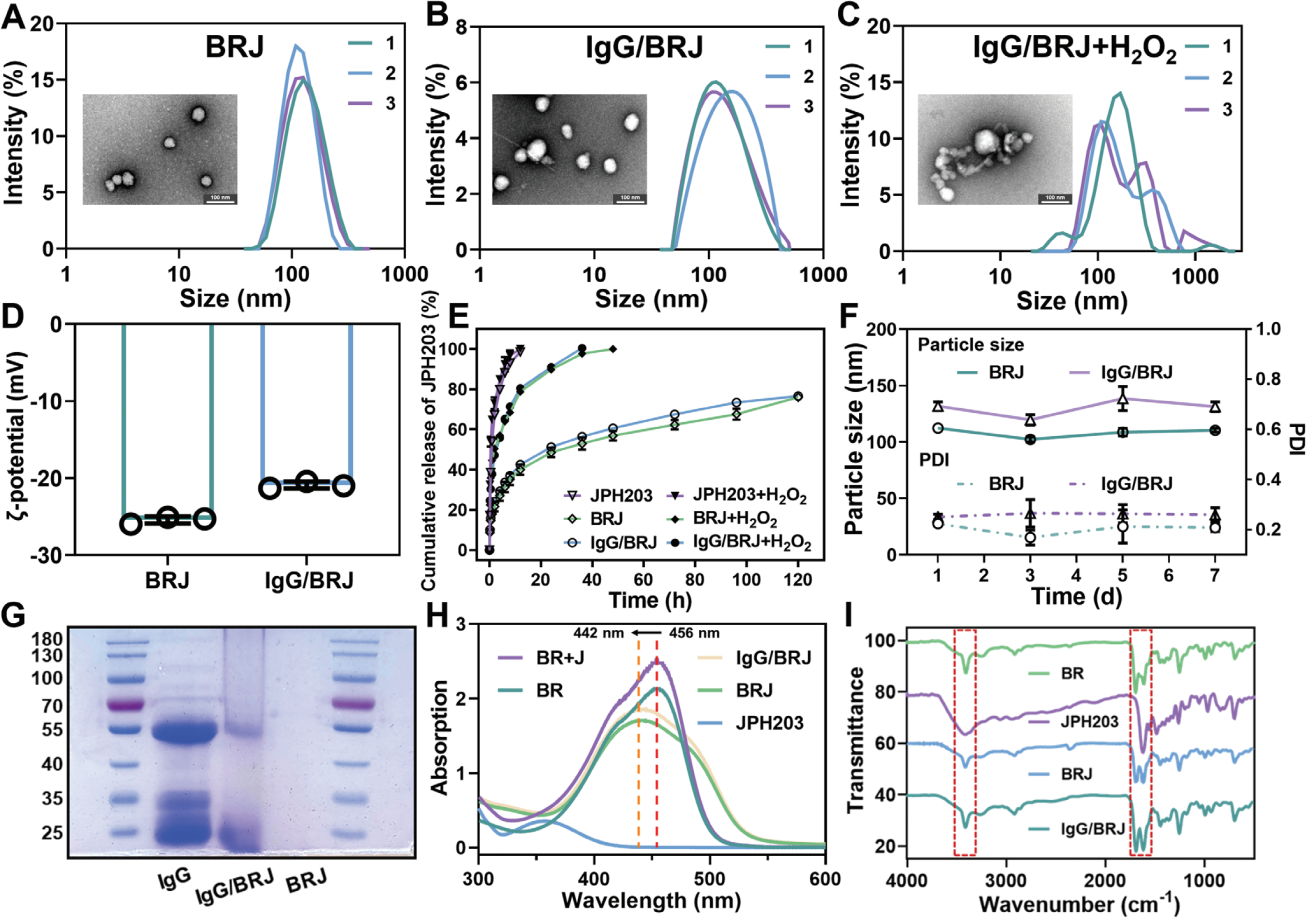
Characterization of nanoparticle IgG/BRJ. Particle size distribution and TEM images of A) BRJ, B) IgG/BRJ, and C) IgG/BRJ in the presence of H_2_O_2_. D) Zeta potential of BRJ and IgG/BRJ. E) In vitro release profile of JPH203 from free JPH203, BRJ, and IgG/BRJ in phosphate‐buffered solution (PBS; pH 7.4) containing 0.5% Tween 80 with or without 0.3% H_2_O_2_. F) Stability of BRJ and IgG/BRJ was investigated by determining the particle size and PDI in 7 days. G) Coomassie Brilliant Blue stain image of IgG, IgG/BRJ, and BRJ in SDS‐PAGE gel. H) UV–vis spectroscopy of BR, JPH203, mixture of BR and JPH203, and BRJ and IgG/BRJ. I) FTIR spectroscopy of BR, JPH203, BRJ and IgG/BRJ. Data in A, B, and C were measured in parallel three times; data in D, E, and F are expressed as mean ± SD (n = 3).

Following a comprehensive investigation, characterization assays of BRJ and IgG/BRJ nanoparticles were performed. The mean diameter of BRJ was 113.9 ± 8.3 nm, whereas IgG/BRJ exhibited a slightly larger size of 126.8 ± 15.1 nm (**Figure** **1**A,B). Transmission electron microscopy (TEM) and scanning electron microscopy (SEM) images (Figure S4, Supporting Information) confirmed the spherical morphology of both nanoparticles, with IgG/BRJ displaying a slightly larger size than BRJ. To assess the oxidative responsiveness of IgG/BRJ, 0.3% H_2_O_2_ was added. Figure 1C presents the particle size distribution of the IgG/BRJ disintegrated under oxidative stress, indicating the responsiveness of the nanoparticles to oxidative conditions. Moreover, TEM images revealed that most of the nanoparticles were disrupted, further confirming the oxidative stress response of IgG/BRJ. Notably, both BRJ and IgG/BRJ exhibited negative and similar zeta potentials (Figure 1D), implying that the addition of IgG minimally influenced the nanoparticle potential. An in vitro release assay of JPH203 from IgG/BRJ under normal and high‐oxidation conditions was performed. The free JPH203 exhibited a rapid release, unaffected by the environment, with a cumulative release rate of 100% after ≈12 h (Figure 1E). In comparison, BRJ and IgG/BRJ demonstrated sustained release profiles in normal release medium, with a cumulative release rate of only 76.62% after 120 h. To simulate the characteristics of the high‐oxidation environment of OA, H_2_O_2_ was added to the release medium. Notably, the release rate of JPH203 from both BRJ and IgG/BRJ was significantly accelerated under high oxidation conditions, with complete release achieved at 48 and 36 h, respectively. These results were consistent with those of the TEM observations, further confirming the ROS‐responsive nature of IgG/BRJ. This could be attributed to the conversion of BR to hydrophilic biliverdin under oxidizing conditions, which disrupts the nanoparticle structure and facilitates drug release. Furthermore, the stability of IgG/BRJ was evaluated by monitoring the nanoparticle size and PDI during a one‐week storage period. The observed fluctuations in both the nanoparticle size and PDI were minimal during the observation period, indicating the excellent stability of BRJ and IgG/BRJ (Figure 1F). To confirm the successful binding of IgG to the BRJ surface, sodium dodecyl sulfate‐polyacrylamide gel electrophoresis (SDS‐PAGE) was performed, and the bands were stained with Coomassie Brilliant Blue. The results demonstrated that IgG/BRJ exhibited protein bands similar to those of IgG alone, whereas these bands were not present in BRJ (Figure 1G). This finding confirmed the successful binding of IgG to the BRJ surface, with a binding ratio of 94.25 ± 2.25%. Furthermore, the interaction between BR and JPH203 in nanoparticles was analyzed using ultraviolet‐visible (UV–vis) spectroscopy. The UV–vis assay revealed a strong absorption peak at 456 nm for BR and 358 nm for JPH203 (Figure 1H). After the formation of the nanostructures, the absorption peaks of BR shifted to 442 nm, whereas the absorption peak of JPH203 disappeared. This shift in the absorption peaks strongly suggests an interaction between BR and JPH203 in BRJ and IgG/BRJ, indicating successful nanostructure formation. Subsequently, Fourier transform infrared spectroscopy (FTIR) analysis was performed (Figure 1I). The hydroxyl group (‐OH) of BR was represented by a characteristic peak at 3420 cm^−1^, whereas JPH203 did not exhibit an obvious characteristic peak at this frequency. However, the curves of BRJ and IgG/BRJ demonstrated clear characteristic peaks at 3420 cm^−1^. Additionally, the C═O band of BR was represented by stronger characteristic peaks at 1700 cm^−1^ and weaker peaks at 1600 cm^−1^, whereas JPH203 had a characteristic peak of the C═O band at 1600 cm^−1^. The intensity of the characteristic peaks at 1700 and 1600 cm^−1^ in the curves of BRJ and IgG/BRJ were similar. These changes in the characteristic peaks within the dashed box indicate interactions between BR and JPH203. However, no new characteristic peaks were observed, suggesting that no new chemical bonds were formed between the two compounds. Moreover, the adsorption of IgG did not significantly affect the peak profile, as observed by UV–vis or FTIR spectroscopy. These results provided a robust foundation for subsequent in vitro and in vivo biological evaluations.

### 2.2 Cytocompatibility and Cell Targeting of IgG/BRJ

**Figure 2:**
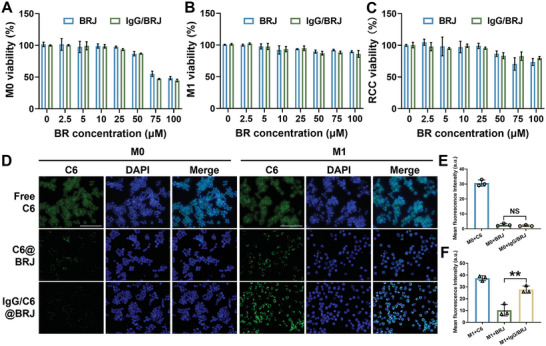
Cell safety and cellular uptake of BRJ and IgG/BRJ. Cell viability of A) M0, B) M1, and C) RCC after treatment with BRJ or IgG/BRJ for 24 h. D) Fluorescence image displaying the uptake features of free C6, C6@BRJ, and IgG/C6@BRJ in M0 and M1. (C6: green; cell nuclei/DAPI: blue). Scale bar = 100 µm. Quantitative analysis of the uptake assay in E) M0 and F) M1. Data are expressed as mean ± SD (n = 3). NS *P* > 0.05, **P < 0.01.

The cytocompatibility of BRJ and IgG/BRJ has been meticulously investigated before conducting in vitro experiments. This assessment was performed using 24 and 48 h methyl thiazolyl tetrazolium (MTT) assays, which allowed us to gauge the impact of these formulations on cellular viability. Specifically, M0, M1, and Rat Cartilage Cells (RCC) were used as representative cell models (**Figure** **2**A–C; Figure S5A–C, Supporting Information). Notably, no significant toxicity was observed in M0 cells, M1 cells, or RCC even with increasing concentrations of BR in BRJ and IgG/BRJ up to 25 µm, and the cell viability rate remained ≈100% under these conditions. However, a minimal inhibitory effect on the proliferation of M0 cells and RCC was observed at 50 µm concentrations of BR in BRJ and IgG/BRJ, and the inhibitory effect increased with increasing concentrations of BR to 75 and 100 µm. However, the cytocompatibility of BRJ and IgG/BRJ remained favorable in M1 cells, even when the BR concentration reached 100 µm. Notably, at these concentrations, both BRJ and IgG/BRJ showed good biosafety in M1 cells. Overall, the MTT assay results suggested that BRJ and IgG/BRJ were characterized by excellent biosafety (≤ 20 µm). These findings underscore the potential of these formulations for various in vitro applications and highlight their promising cytocompatibility with different cell types, which is pivotal for their potential use in diverse biological contexts.

In this study, an opsonized nanoparticle system was engineered to selectively induce recognition and uptake by M1 macrophages, thereby achieving targeted regulation of macrophages M1‐M2. Successful internalization of these nanoparticles by M1 macrophages is essential to trigger the repolarization of M1 to M2 macrophages. To study the specific targeting ability of IgG/BRJ to M1 macrophages, a cell uptake assay using coumarin 6 (C6) as the probe was performed. The free C6 exhibited strong fluorescence intensity in both M0 and M1 cells, which can be attributed to its hydrophobic nature (Figure 2D). The fluorescence intensities of C6‐labeled IgG/BRJ (IgG/C6@BRJ) and C6‐labeled BRJ (C6@BRJ) in M0 cells were comparable (Figure 2E). This observation is consistent with the relatively low phagocytic capacity of macrophages before activation. However, upon activation of M0 to M1, the phagocytic capacity was significantly enhanced, and the high expression of FcγRs receptors on the M1 membrane facilitated the phagocytosis of IgG‐conjugated particles.^[^ ^34^ ^]^ Consequently, a discernible enhancement in the fluorescence intensities of free C6 and C6@BRJ in M1 cells was observed (≈1.2 times and 3.7 times, respectively). Notably, the fluorescence intensity of IgG/C6@BRJ increased by ≈12.9 times, significantly higher than that of other tested formulations (Figure 2F). This outcome indicates that binding with IgG could effectively promote the uptake of nanoparticles by M1 cells. These results elucidate the specific targeting effect of IgG/BRJ on M1 cells, making it a promising drug candidate for targeted delivery and treatment of OA. The effective uptake of IgG‐conjugated nanoparticles by activated M1 macrophages opens up promising prospects for precise regulation of macrophage polarization and holds potential implications for targeted therapeutic interventions in OA.

### 2.3 In Vitro M1‐M2 Macrophage Repolarization Study

**Figure 3:**
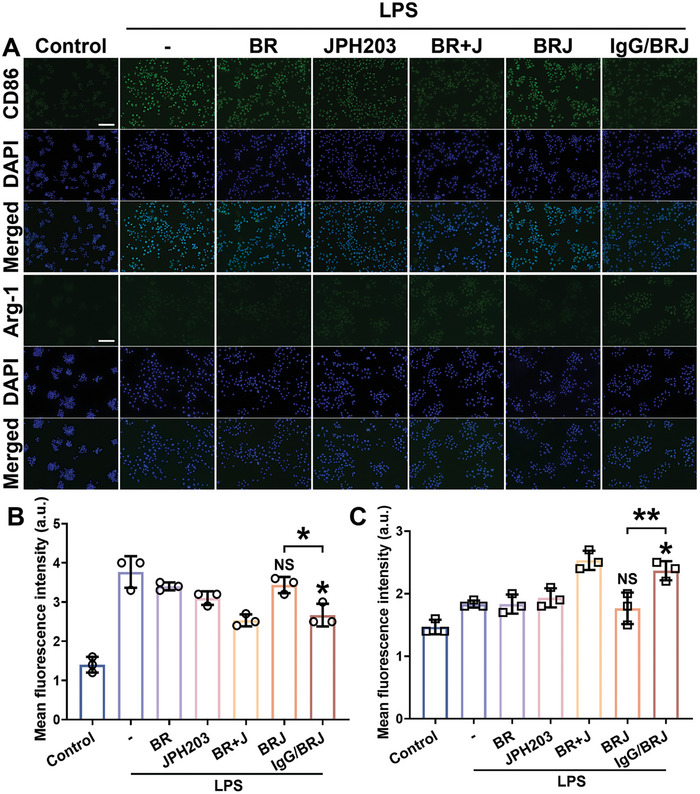
IgG/BRJ promotes M1 to M2 polarization of macrophages. A) After different treatments, the expressions of M1 biomarker CD86 and M2 biomarker Arg‐1 were detected by IF assay, and the nuclear locations were marked by DAPI. Scale bar = 100 µm. B) Quantitative analysis of the fluorescence intensity of CD86 expression. C) Quantitative analysis of the fluorescence intensity of Arg‐1 expression. Data in B and C are expressed as mean ± SD (n = 3), NS *P* > 0.05, *P < 0.05, **P < 0.01, compared to the LPS group or as indicated.

M1 macrophages play a pivotal role in fostering an inflammatory microenvironment and OA progression by producing excessive inflammatory cytokines and mediators. Conversely, M2 macrophages exhibit anti‐inflammatory properties that impede OA progression. Consequently, facilitation of M1 macrophage repolarization into M2 macrophages represents a promising therapeutic strategy for OA treatment. Herein, we first assessed the in vitro potential of IgG/BRJ to induce the polarization of macrophages from the M1 to M2 phenotype using immunofluorescence (IF) analysis with CD86 and Arg‐1 as biomarkers for M1 and M2, respectively. A notable increase in CD86 fluorescence intensity following LPS stimulation was observed, accompanied by a slight enhancement in Arg‐1 fluorescence intensity (**Figure** **3**A). Treatment with BR or JPH203 alone exerted only minimal effects. However, in the combined BR and JPH203 treatment and the IgG/BRJ groups, CD86 fluorescence intensity was significantly attenuated, whereas Arg‐1 fluorescence intensity was substantially enhanced. Notably, the M2 polarization effect of BRJ was weaker than that of IgG/BRJ, similar to the effect observed in the BR and JPH203 groups. The uptake by BRJ was lower than that of IgG/BRJ, resulting in a weaker function, whereas the combination of free BR and JPH203 could directly interact with the cells, demonstrating a stronger effect than did BRJ (Figure 2D‐F). These findings were validated by quantification (Figure 3B,C). The results of flow cytometry further validated and confirmed the ability of IgG/BRJ to regulate macrophage repolarization from M1 to M2 in vitro (Figure S6, Supporting Information).

### 2.4 Mechanisms Underlying M1 to M2 Repolarization by IgG/BRJ

**Figure 4:**
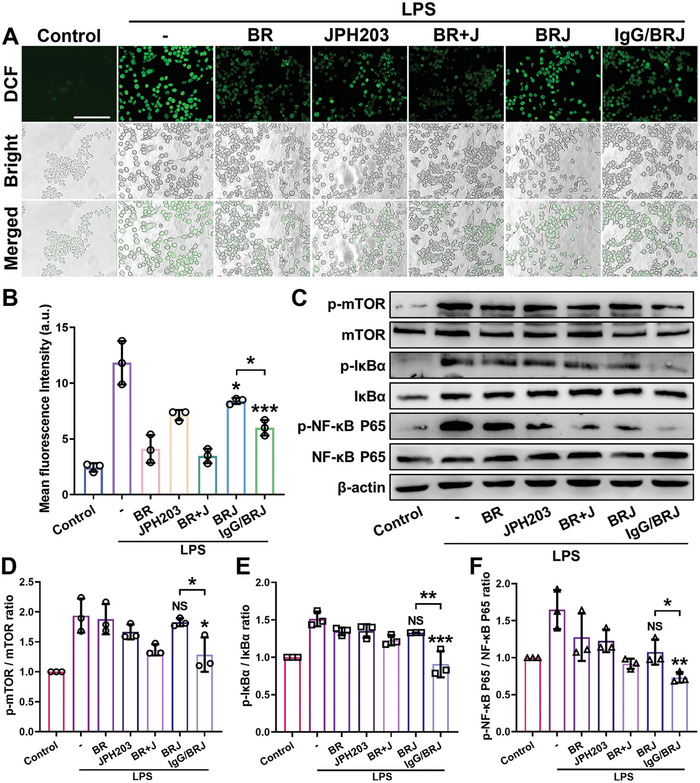
Mechanism of IgG/BRJ regulated macrophage polarization. A) ROS levels in RAW 264.7 cells after different treatments were detected by the DCFH‐DA probe. Scale bar = 100 µm. B) DCF fluorescence quantitative analysis. C) Expression of p‐mTOR, mTOR, p‐IκBα, IκBα, p‐NF‐κB P65, and NF‐κB P65 in RAW 264.7 after LPS stimulation and treatment with different formulations. D–F) Quantification analysis of (C). Data are expressed as mean ± SD (n = 3), NS *P* > 0.05, *P < 0.05, **P < 0.01, ***P < 0.001, compared to the LPS groups or as indicated.

Given the ability of IgG/BRJ to induce M1‐M2 polarization, our investigation further delineated the underlying regulatory mechanisms. Elevated ROS levels are a key pathological characteristic of OA.^[^ ^35^ ^]^ ROS can facilitate M1 macrophage polarization, consequently exacerbating synovitis, extracellular matrix (ECM) degradation, and subchondral bone dysfunction.^[^ ^36^ ^]^ Additionally, the mTOR and NF‐κB signaling pathways play crucial roles in modulating M1 cell polarization.^[^ ^22^, ^37^ ^]^ Because the opsonized nanoparticle IgG/BRJ primarily comprised the endogenous antioxidant BR and the mTOR pathway inhibitor JPH203, we postulated that IgG/BRJ promotes M1‐M2 polarization by effectively scavenging ROS and inhibiting the mTOR and NF‐κB pathways. To assess ROS levels, a DCFH‐DA fluorescent probe was used to observe a robust green DCF signal in macrophages following LPS stimulation, which indicated a substantial increase in ROS levels in M1 macrophages. Among the treatment groups, BR and BR+J exhibited the most potent ROS elimination effects, whereas JPH203 also demonstrated some inhibitory capabilities. Interestingly, ROS fluorescence after IgG/BRJ treatment was notably weaker than that in the BRJ treatment group, indicating the superior ROS clearance ability of the IgG/BRJ formulation. Quantitative analysis of ROS levels further substantiated these observations (**Figure** **4**A,B). The expression levels of mTOR and NF‐κB signaling pathway components in the M1 macrophages were evaluated using western blot (WB) analysis. Compared to the control group, the expressions of p‐mTOR, p‐NF‐κB P65, and p‐IκBα were significantly elevated in M1 macrophages, indicative of pathway activation. However, upon treatment with IgG/BRJ, the expression levels of p‐mTOR, p‐NF‐κB P65, and p‐IκBα were notably reduced in M1 macrophages (Figure 4C–F). Quantitative analysis of the WB results aligned with the trends observed in ROS measurements, elucidating that IgG/BRJ is capable of regulating M1‐M2 macrophage polarization via ROS clearance and inhibition of mTOR and NF‐κB signaling pathways. Furthermore, these findings are consistent with the M1‐M2 polarization function of IgG/BRJ, as previously demonstrated in Figure 3.

### 2.5 Metabolic Regulation of IgG/BRJ on M1

**Figure 5:**
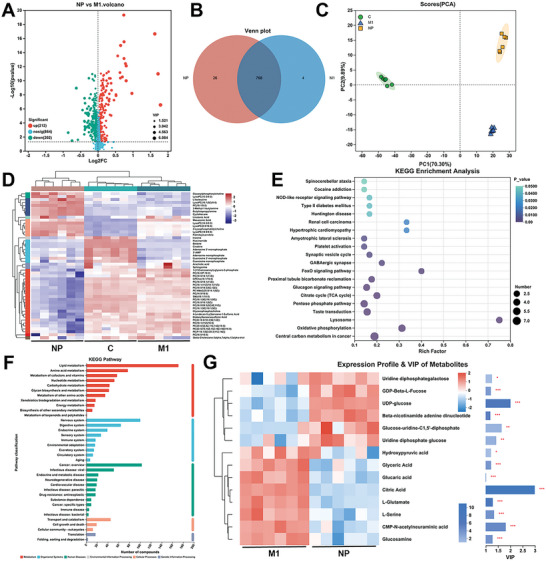
In vitro metabolic regulation effects of IgG/BRJ in RAW 264.7 cells. A) Volcano plot of the differentially expressed metabolites in RAW 264.7 between the control and LPS‐treated groups. Red and green dots indicate significantly up‐ or down‐regulated metabolites, respectively (*P* < 0.05 and VIP > 1). B) Venn diagram of metabolite expression in RAW 264.7 between the control, M1, and IgG/BRJ‐treated (NP) group. C) 2D principal component analysis (PCA) score plots in RAW 264.7 between the control, M1, and IgG/BRJ‐treated (NP) group. D) Metabolite clustering analysis in RAW 264.7 between the control, M1, and IgG/BRJ‐treated (NP) group. E) KEGG pathway enrichment analysis, *P* < 0.05. F) KEGG functional pathway analysis. G) VIP analysis in RAW 264.7 between the M1 and IgG/BRJ‐treated (NP) group (*P < 0.05, **P < 0.01, ***P < 0.001, and VIP > 1).

As previously described, IgG/BRJ can modulate M1‐M2 macrophage polarization through the inhibition of the mTOR and NF‐κB pathways and the scavenging of ROS. Given the close association between the mTOR pathway and organismal metabolic activity,^[^ ^38^ ^]^ a metabonomic analysis was performed to determine whether IgG/BRJ influences macrophage polarization by influencing macrophage metabolism. The analysis identified 1315 metabolites in the macrophage samples, among which 212 exhibited a significant increase and 202 metabolites displayed a notable decrease in the IgG/BRJ‐treated (NP) group compared with the M1 group (**Figure** **5**A). The Venn diagram illustrates 768 shared metabolites between the IgG/BRJ‐treated and untreated M1 groups, of which 26 and 4 were specific to the NP and M1 groups, respectively (Figure 5B). Principal component analysis (PCA) indicated that the coordinates for the control, M1, and NP groups were distinctly separated, signifying substantial distinctions among these three groups and underscoring that IgG/BRJ treatment considerably influenced macrophage metabolism (Figure 5C). Metabolites with similar expression patterns were considered functionally significant. Therefore, the metabolites in the control, M1, and NP groups were subjected to metabolite clustering analysis (Figure 5D), with outcomes similar to those observed in PCA. This further highlights the considerable disparity in the metabolism of macrophages in the IgG/BRJ‐treated group compared with that in the control and M1 groups. Subsequently, Kyoto Encyclopedia of Genes and Genomes (KEGG) enrichment and functional pathway analysis were used to identify the prominent energy metabolism, inflammatory regulation, and biosynthesis pathways (Figure 5E,F). Variable importance in projection (VIP) value analysis demonstrated that citric acid substantially contributed to the IgG/BRJ‐induced alterations in macrophage metabolic function (Figure 5G). The activation of the tricarboxylic acid cycle (TCA cycle) can attenuate the pro‐inflammatory effects of M1 macrophages,^[^ ^39^ ^]^ suggesting that IgG/BRJ may stimulate the TCA cycle and facilitate macrophage polarization toward the M2 phenotype. Quantitative analysis of metabolites in the M1, M2, and NP groups revealed a significant disparity in citric acid metabolism between macrophages treated with IgG/BRJ and M1 macrophages, similar to that in M2 macrophages (Figure S7A, Supporting Information). Although succinic acid abundance was not significantly different between the IgG/BRJ, M1, and M2 groups, the ratio of succinic acid to citric acid was markedly higher in the IgG/BRJ group than in the M1 group. This implied a greater conversion of citric acid to succinic acid (Figure S7B,C, Supporting Information), indicating an activated TCA cycle. Furthermore, quantitative assessment of coenzyme A abundance demonstrated a substantial increase after IgG/BRJ treatment, aligning more closely with the levels observed in M2 macrophages than in M1 (Figure S7D, Supporting Information). Elevated coenzyme A levels are indicative of enhanced TCA cycle activity, as numerous reactions in the TCA cycle require the involvement of coenzyme A.^[^ ^40^ ^]^ Therefore, IgG/BRJ can activate the TCA cycle within macrophages and promote their polarization toward the M2 phenotype. Moreover, IgG/BRJ decreased the abundance of metabolites associated with the NF‐κB pathway (Figure S7E, Supporting Information), corroborating the results of WB analysis (Figure 4C). Given that low oxygen levels and elevated ROS are characteristic pathological features in OA,^[^ ^41^ ^]^ along with the activation of hypoxia‐inducible factor‐1α (HIF‐1α) that regulates the activation and pro‐inflammatory responses of M1 macrophages,^[^ ^16^ ^]^ the abundance of metabolites linked to the HIF‐1α pathway was significantly decreased in the IgG/BRJ‐treated group than in the M1 group (Figure S7F, Supporting Information). This could be primarily attributed to the efficacy of IgG/BRJ in ROS clearance. Consequently, metabolomic analysis highlighted that IgG/BRJ could stimulate the TCA cycle, facilitating the regulation of M1‐M2 macrophage polarization with concurrent inhibition of the NF‐κB and HIF‐1α pathways.

### 2.6 In Vitro Anti‐Inflammatory Effects of IgG/BRJ

WB was performed to assess the anti‐inflammatory function of IgG/BRJ in M1 macrophages. Compared to the untreated control, pro‐inflammatory markers (TNF‐α, IL‐1β, and IL‐6) were significantly upregulated in the LPS‐stimulated group, whereas the anti‐inflammatory marker (IL‐10) remained relatively constant (Figure S8A, Supporting Information). Upon treatment with BR or JPH203, the expression level of IL‐6 declined more significantly than that of TNF‐α and IL‐1β, and IL‐10 levels increased obviously. Notably, the anti‐inflammatory effects of BRJ on M1 macrophages were comparable to those of BR or JPH203 alone, which were consistent with the results from other experiments owing to an insufficient uptake. Interestingly, combination treatment of BR with JPH203 and IgG/BRJ demonstrated a robust inhibition of pro‐inflammatory markers and significantly upregulated anti‐inflammatory marker expression. The enhanced uptake of IgG/BRJ by M1 macrophages effectively performed the functions of BR and JPH203. The quantitative analysis further corroborated and validated the WB results, elucidating the excellent anti‐inflammatory effects of IgG/BRJ in vitro (Figure S8B–E, Supporting Information).

### 2.7 In Vitro Protection Effect of IgG/BRJ on RCC

The ability of IgG/BRJ to regulate M1‐M2 polarization has been previously reported. To assess the protective function of IgG/BRJ on chondrocytes in the context of the OA environment, different conditioned media of RAW 264.7 cells were collected to simulate the environment in which chondrocytes reside, and to evaluate the potential of IgG/BRJ to safeguard chondrocytes (RCC). ROS levels in RCC were initially measured after various treatments using DCFH‐DA. The control and M0M groups exhibited extremely weak green fluorescence intensities, whereas the M1M group demonstrated the strongest signal, indicating high ROS levels (Figure S9A, Supporting Information). Notably, among all the test groups, ROS production was significantly reduced in LPS‐induced RAW 264.7 cells treated with IgG/BRJ. The quantitative ROS data further confirmed these observations (Figure S10, Supporting Information), suggesting that IgG/BRJ can effectively suppress ROS production in RCC by inhibiting ROS production in M1 macrophages and promoting M2 polarization.

The inflammatory state of RCC using WB was then investigated, focusing on the secretion of inflammatory factors and the activation status of the NF‐κB pathway. RCC cultured with M1M exhibited a significant upregulation in the expression of pro‐inflammatory factors (IL‐1β, IL‐6, and TNF‐α) and an activated NF‐κB pathway (p‐P65, p‐IκBα) (Figure S9B, Supporting Information). Conversely, RCC cultured in M1M‐5 (treated with IgG/BRJ) exhibited a substantial reduction in pro‐inflammatory factor expression and NF‐κB pathway suppression. Although the other treatments showed a decreasing trend to some extent, they were less effective than IgG/BRJ. Consistent with those of the quantitative analysis findings, IgG/BRJ treatment can mitigate the inflammatory response in RCC by promoting M1‐M2 polarization and ameliorating the inflammatory environment (Figure S9C–G, Supporting Information). These findings highlight the potential of IgG/BRJ as a therapeutic candidate for attenuating inflammation in patients with OA.

**Figure 6:**
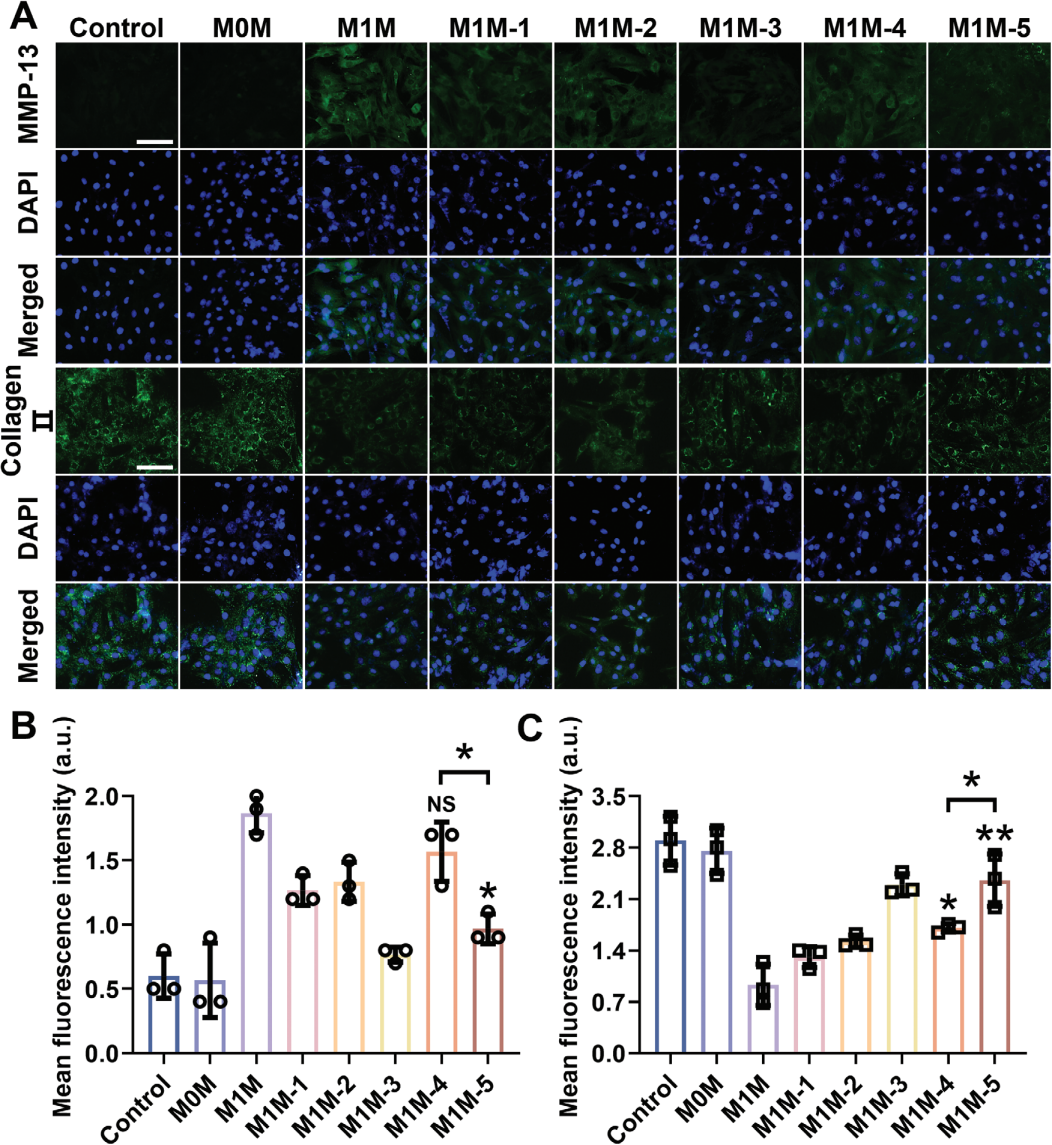
IgG/BRJ enhances anabolism level of RCC. A) MMP‐13 and collagen II IF images of RCC cultured in different conditional medium. Scale bar = 100 µm. B) Quantitative analysis of the fluorescence intensity of MMP‐13. C) Quantitative analysis of the fluorescence intensity of collagen II. Data are expressed as mean ± SD (n = 3). NS *P* > 0.05, *P < 0.05, **P < 0.01, compared to M1M group or as indicated. Control: normal RCC; M0M: RCC cultured in M0 conditioned medium culture; M1M: RCC cultured without treatment in M1 conditioned medium; M1M‐1: RCC cultured in BR‐treated M1 conditioned medium; M1M‐2: RCC cultured in JPH203‐treated M1 conditioned medium; M1M‐3: RCC cultured in BR+J‐treated M1 conditioned medium; M1M‐4: RCC cultured in BRJ‐treated M1 conditioned medium; M1M‐5: RCC cultured in IgG/BRJ‐treated M1 conditioned medium.

Furthermore, the inflammatory environment driven by M1 macrophages can disrupt chondrocyte metabolism, leading to the aggravation of catabolic processes. This exacerbates the inflammatory responses and accelerates OA progression. Therefore, elevating anabolic levels in chondrocytes is crucial for OA treatment. To assess the metabolic activity of RCC, an IF assay was performed using matrix metalloproteinase (MMP)‐13 as an indicator of catabolism and collagen II as an indicator of anabolism. The MMP‐13 signal was weaker in the control and M0M groups, whereas the collagen II signal was strong, indicating that the RCC was in a healthy state (**Figure** **6**A). However, upon culturing in the M1M environment, the fluorescence intensity of MMP‐13 increased and the fluorescence intensity of collagen II decreased significantly, indicating the disruption of metabolic homeostasis in RCC and the transition of chondrocytes into a pathological state resembling OA. In contrast, compared with the other treatment groups, MMP‐13 fluorescence significantly decreased in the M1M‐5 group, with a notable increase in collagen II fluorescence intensity. These findings were validated by the quantitative fluorescence analysis (Figure 6B,C). The IF assay indicated that IgG/BRJ effectively inhibited the expression of catabolic enzymes and protected RCC. In addition, the expression levels of MMP‐13 and collagen II were analyzed using immunoblotting. The results of the immunoblot analysis were consistent with the trends observed in the IF analysis, further confirming the ability of IgG/BRJ to modulate chondrocyte metabolism and protect RCC from catabolic processes associated with OA pathology (Figure S11, Supporting Information). These findings highlight the potential of IgG/BRJ as a promising therapeutic agent for mitigating OA progression by restoring chondrocyte anabolism and inhibiting catabolism.

In vitro experimental results clearly demonstrated the direct and multifaceted effects of IgG/BRJ in mitigating the inflammatory response induced by M1 macrophages. By modulating the inflammatory response and inhibiting catabolic processes, IgG/BRJ acts as a protective agent for chondrocytes, preserving their metabolic homeostasis and promoting anabolic activities. IgG/BRJ contributes significantly to the protection and repair of chondrocytes, and facilitates the improvement and remodeling of the chondroinflammatory microenvironment. This has potential implications for the improvement and regeneration of the ECM in the affected cartilage.

### 2.8 In Vivo Therapeutic Efficacy and Underlying Mechanisms of IgG/BRJ on OA

**Figure 7:**
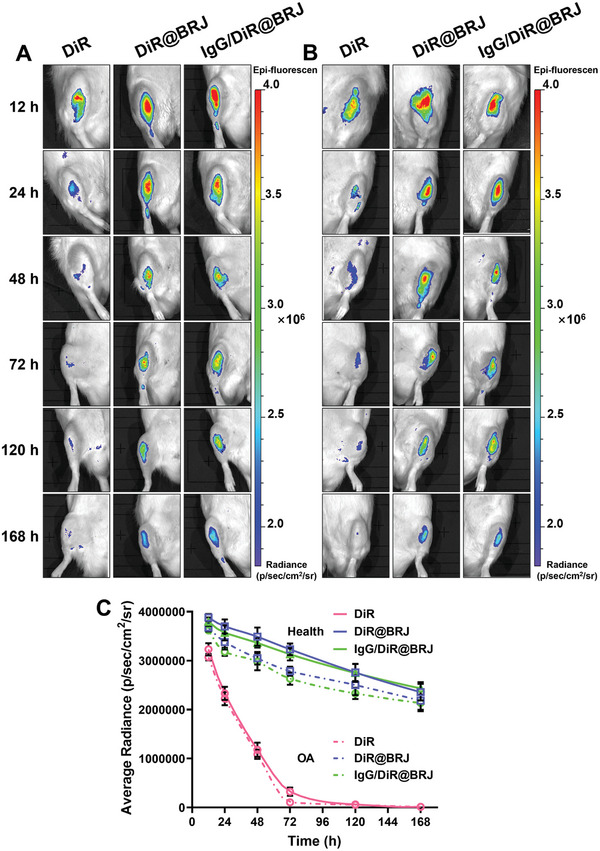
In vivo imaging after IgG/DiR@BRJ administration into healthy or OA rat knees. The whole process of the retention of nanoparticles in vivo was tracked by DiR at 12, 24, 48, 72, 120, and 168 h after administration. A) OA knees. B) Healthy knees. C) Quantification analysis of fluorescence intensity in healthy or OA knees at different time periods. Data are expressed as mean ± SD (n = 3).

To test the in vivo therapeutic efficacy of IgG/BRJ, nanoparticle retention after intra‐articular injection was assessed using 1, 1′‐dioctadecyl‐3, 3, 3′, 3′‐tetramethylindotricarbocyanine iodide (DiR), a near‐infrared dye, to trace different formulations, including free DiR, DiR@BRJ, and IgG/DiR@BRJ, using an in vivo imaging system (IVIS). The in vivo behavior of the nanoparticles was compared in both OA (**Figure** **7**A) and healthy joints (Figure 7B). The results indicated that free DiR showed a substantial decrease in the signal 24 h after intra‐articular injection in both healthy and OA joints. This rapid signal decline is consistent with those of previous reports, as small molecules are typically excreted swiftly through lymphatic vessels or capillaries.^[^ ^25^, ^42^ ^]^ In contrast, the DiR@BRJ and IgG/DiR@BRJ groups demonstrated better retention after intra‐articular injection for 24 h till 168 h than did free DiR. Interestingly, the fluorescence signal diminished more rapidly in OA joints between 24 and 120 h than in healthy joints (Figure 7C). This difference was attributed to the increased ROS levels in the OA environment, which expedited the release of the nanoparticle drugs (Figure 1E). Furthermore, no significant difference in the retention effect within the joint cavity was observed between the DiR@BRJ and IgG/DiR@BRJ groups after drug administration. This suggests that IgG binding plays a limited role in prolonging the bioretention of nanoparticles in the joint. Overall, the findings indicated that the DiR@BRJ and IgG/DiR@BRJ formulations exhibited improved retention properties after intra‐articular injection, making them suitable candidates for further in vivo therapeutic evaluation.

**Figure 8:**
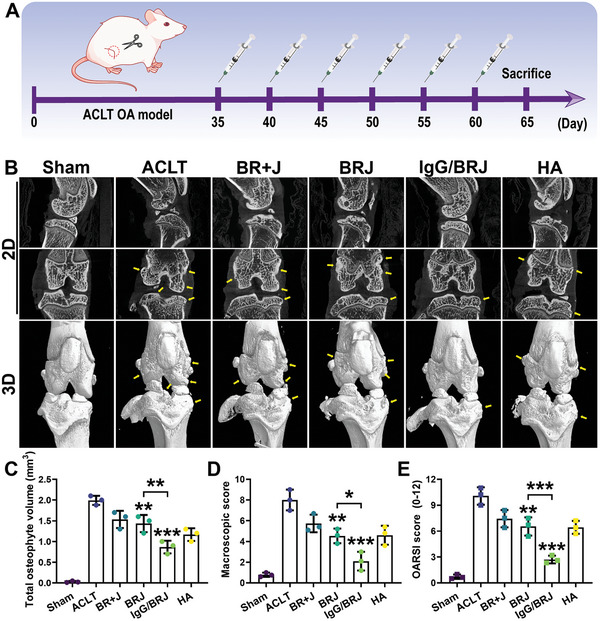
In vivo therapeutic effects of IgG/BRJ in OA models. A) In vivo experimental design. B) 2D and 3D macro‐CT images of rat knees (osteophytes indicated by yellow arrows). C) Quantification of total osteophyte volume in the knee joint of rats. D) Macroscopic score of cartilage. E) OARIS score of cartilage. Data are expressed as mean ± SD (n = 3). *P < 0.05, **P < 0.01, ***P < 0.001, compared to the ACLT group or as indicated.

For subsequent in vivo evaluation, anterior cruciate ligament transection (ACLT) rats were used to assess the therapeutic effect of IgG/BRJ. The rats were randomly divided into six groups on the 35th postoperative day, with sham‐operated rats serving as healthy controls. The ACLT group received intra‐articular injections of saline, BR+J, BRJ, IgG/BRJ, and hyaluronic acid (HA) into the right knee every 5 days. HA was used as a positive control from a clinical perspective. On the 65th day, the rats were euthanized, and their joints were collected for further experiments (**Figure** **8**A). As shown in Figure S12a,b (Supporting Information), severe joint swelling was observed in the ACLT group compared to the sham‐operated group. However, the drug treatment groups demonstrated a certain degree of improvement in joint swelling. Following joint dissection to expose the articular cavity (Figure S12c, Supporting Information), the cartilage exhibited severe defects in the ACLT group. In contrast, the IgG/BRJ treatment group displayed a significant improvement in cartilage defects, and the cartilage surface was noticeably smoother, resembling that in the sham‐operated group. Although the other treatment groups showed some efficacy, they were not as effective as IgG/BRJ in preserving cartilage integrity.

To further assess osteophyte and subchondral bone damage in the knee joint after ACLT, micro‐computed tomography (Micro‐CT) scanning was performed. The 2D sagittal and coronal scanned images of the knee joint revealed osteophytes marked with yellow arrows (Figure 8B). In the ACLT group, micro‐CT images revealed a noticeable increase in osteophyte formation along with the presence of severe pits and cracks on the surface, indicating significant cartilage and subchondral bone damage. In contrast, the IgG/BRJ treatment group exhibited the best efficacy, with minimal osteophytes and intact subchondral bone. This observation was further confirmed by quantifying the total osteophyte volume (Figure 8C) and the macroscopic score (Figure 8D), demonstrating that IgG/BRJ can effectively protect the cartilage and delay the progression of OA.

**Figure 9:**
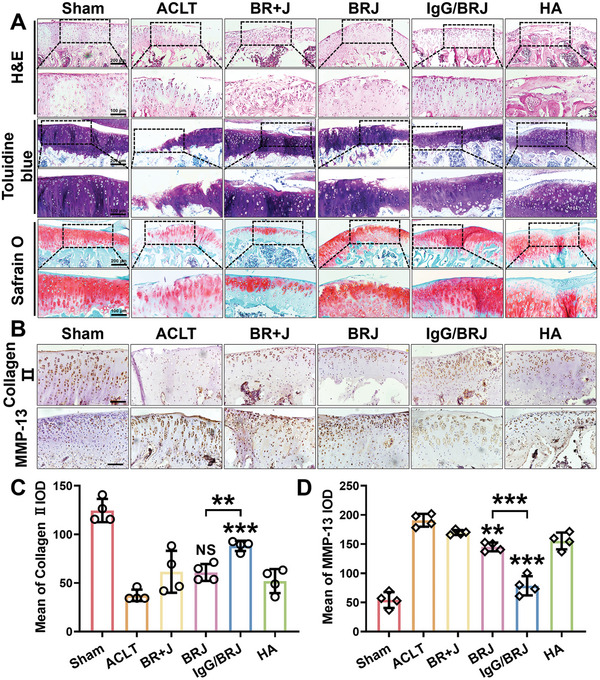
Effects of IgG/BRJ on cartilage repair. A) Knee cartilage sections stained with H&E, toluidine blue, and safranin O. B) IHC assay of collagen II and MMP‐13 in knee cartilage. Scale bar = 100 µm. C,D) Quantitative analysis of (B). Data are expressed as mean ± SD (n = 4). NS *P* > 0.05, **P < 0.01, ***P < 0.001, compared to the ACLT group or as indicated.

The cartilage condition in OA after treatment was evaluated using hematoxylin‐eosin (H&E) staining, safranin O and fast green staining, and toluidine blue staining to histologically assess the cartilage tissue. The ACLT group exhibited the most severe cartilage destruction, which was characterized by surface unevenness, cell degeneration, and ECM degradation (**Figure** **9**A). By contrast, the sham‐operated group had a smooth cartilage surface. Notably, the severely damaged cartilage became smoother and more structurally intact after IgG/BRJ treatment, indicating their potential cartilage repair capabilities. safranin O‐fast green and toluidine blue staining further confirmed the extent of cartilage damage in the ACLT group, where glycosaminoglycans (GAGs) were distributed unevenly and significantly reduced. Among the treated OA rat groups (BR+J, BRJ, and HA), some degree of cartilage damage reduction was observed, with the IgG/BRJ group displaying the highest GAGs content, which was comparable to that of the sham‐operated group. Additionally, the Osteoarthritis Research Society International (OARSI) score (Figure 8E) demonstrated that the IgG/BRJ group exhibited the most favorable treatment effect, with the lowest score among the five ACLT groups.

Furthermore, to assess metabolic activity in cartilage, an immunohistochemistry (IHC) assay was conducted to measure the levels of collagen II and MMP‐13. The sham‐operated group exhibited low levels of MMP‐13 and high levels of collagen II, indicative of a healthy metabolic condition (Figure 9B–D). In contrast, the ACLT group showed elevated MMP‐13 expression and reduced collagen II expression, indicating the dominance of catabolism in the cartilage. However, after treatment with IgG/BRJ, the high expression of MMP‐13 in the cartilage was reduced, whereas the level of collagen II increased significantly, suggesting a shift in the metabolic activity of the cartilage toward a more normal state.

**Figure 10:**
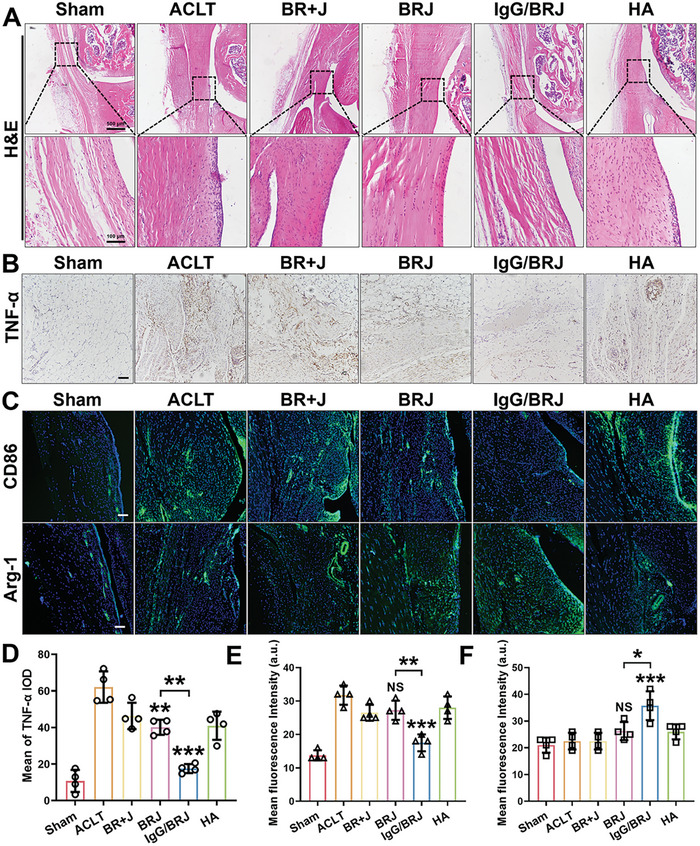
Histological analysis of synovium after different treatments. A) H&E staining of the synovium. The black frame magnifies the inflammatory cells in the synovial area. B) IHC assay of synovium after different treatments. Scale bar = 100 µm. C) IF assay of CD86 and Arg‐1 in the synovium. Scale bar = 100 µm. D) Quantitative analysis of (B). E,F) Quantitative analysis of fluorescence intensity in (C). Data are expressed as mean ± SD (n = 4). NS *P* > 0.05, *P < 0.05, **P < 0.01, ***P < 0.001, compared to the ACLT group or as indicated.

The intricate association between synovitis and the progression and pathogenesis of OA has been recently documented;^[^ ^43^ ^]^ therefore, a histological evaluation of the synovium was performed. H&E staining revealed that the synovial structure was severely disrupted and thickened in the ACLT group, with notable infiltration of inflammatory cells (**Figure** **10**A). However, treatment with IgG/BRJ ameliorated this effect by promoting the repair of the reticular structure of the synovial tissue and inhibiting the infiltration of inflammatory cells. To further assess the inflammatory state of the synovium, an IHC assay was conducted to evaluate TNF‐α expression. TNF‐α expression was remarkably upregulated in the ACLT group (Figure 10B,D). Nevertheless, this upregulation was reversed to varying degrees after administering different formulations, with the most pronounced inhibition observed in the IgG/BRJ treatment group. The results of the H&E staining and IHC demonstrated that IgG/BRJ significantly suppressed synovial inflammation. To investigate the modulatory effect of IgG/BRJ on the macrophage phenotype, IF experiments were conducted using two indicators, CD86 (M1 biomarker) and Arg‐1 (M2 biomarker), to analyze the types of macrophages infiltrating the OA synovium (Figure 10C). Quantitative analysis (Figure 10E,F) revealed a significant increase in the fluorescence signal intensity of CD86 in the ACLT group, indicating the predominance of M1 macrophages. Conversely, the fluorescence signal intensity of Arg‐1 did not differ significantly from that of the sham group, suggesting the limited presence of M2 macrophages. However, after treatment with IgG/BRJ, the fluorescence signal intensity of CD86 was notably reduced, whereas that of Arg‐1 increased significantly. These findings indicate that the administration of IgG/BRJ facilitates the conversion of M1 macrophages into M2 macrophages in the OA synovium. Although IgG binding may have a limited effect on improving the retention capacity of nanoparticles, the use of IgG/BRJ induces active uptake by M1 macrophages, providing an optimal M1‐M2 polarization effect. These experimental results support and validate the effectiveness of IgG/BRJ in regulating macrophage polarization and underscore its potential as a promising therapeutic approach for OA treatment. Furthermore, staining of the rat organs revealed no distinguishable differences between the IgG/BRJ and sham groups, indicating the absence of any potential in vivo toxicity associated with the treatment (Figure S13, Supporting Information).

Collectively, these results demonstrate that intervention with IgG/BRJ can promote cartilage repair and regeneration by regulating M1‐M2 macrophage polarization, making it an effective and promising option for the treatment of OA. Histological and immunohistochemical evidence further supports the potential of IgG/BRJ as a therapeutic candidate for alleviating the pathological changes associated with OA and fostering cartilage restoration.

## 3 Conclusion

Herein, we successfully developed an opsonized bilirubin/JPH203 self‐assembled nanoparticle, IgG/BRJ, which demonstrates promising applications in the treatment of OA. IgG/BRJ exhibited excellent stability and biocompatibility, and its simple preparation method rendered it a practical and feasible therapeutic option. In vitro studies have elucidated the function of IgG/BRJ in inducing phagocytosis by M1 macrophages, which facilitates the repolarization of these macrophages from a pro‐inflammatory M1 phenotype to an anti‐inflammatory M2 phenotype. This effect was particularly beneficial, as it protected chondrocytes from inflammation‐induced damage. Furthermore, our findings revealed that IgG/BRJ exerts its regulatory function on macrophage polarization by scavenging ROS and inhibiting the NF‐κB and mTOR pathways. These mechanisms provide valuable insights into the mode of action by which IgG/BRJ modulates macrophage polarization and attenuates inflammation. In vivo studies in OA rats demonstrated the therapeutic potential of IgG/BRJ. IgG/BRJ increases the number of M2 macrophages in the synovium, thereby effectively reversing the OA‐inflammatory environment and significantly promoting cartilage repair. In conclusion, the development of opsonized IgG/BRJ nanoparticles provides a novel and promising therapeutic strategy for OA. Its ability to modulate macrophage polarization, protect chondrocytes, and promote cartilage repair underscores its potential as an innovative therapeutic strategy for treating OA.

## 4 Experimental Section

### Materials

JPH203 was procured from AZBIOCHEM Co., Ltd. (Shanghai, China). BR was purchased from Macklin Biochemical Co., Ltd. (Shanghai, China). HA with an average molecular weight of 1.3 × 10^6^ Da was obtained from A.V.T Pharmaceutical Tech Co., Ltd. (Shanghai, China). Dialysis bags (MWCO: 3000 Da) were purchased from Shanghai Yuan Ye Biotechnology Co., Ltd. (Shanghai, China). Antifade mounting medium containing DAPI, ROS assay kit, bicinchoninic acid (BCA) protein assay kit, and chemiluminescence (ECL) detection kit were obtained from Beyotime Biotechnology Co., Ltd. (Shanghai, China). IgG from mouse serum (MB2963) and DiR were obtained from Dalian Meilun Biotechnology Co., Ltd. (Dalian, China). MTT, Modified Saffron‐O and Fast Green stain kit, Toluidine Blue stain kit, H&E stain kit, and EDTA decalcifying solution were purchased from Beijing Solarbio Science & Technology Co., Ltd. (Beijing, China). Cell culture grade 1×PBS was obtained from Servicebio (Wuhan, China). Dulbecco's modified Eagle's medium (DMEM), Dulbecco's modified Eagle's medium F‐12 (DMEM/F12), six‐well plates, 96‐well plates, cell culture dishes, and centrifuge tubes were purchased from NEST Biotechnology (Wuxi, China). Immunohistochemistry kits were obtained from Beijing ZSGB Biotechnology Co. Ltd. (Beijing, China). The antibodies against mTOR (2983) and p‐mTOR (5536) were purchased from Cell Signaling Technology (Danvers, MA, USA). Antibodies against CD86 (A1199) were purchased from ABclonal Technology Co., Ltd. (Hubei, China). Antibodies against IL‐10 (orb319439) were obtained from Biorbyt Ltd. (Cambridge, UK). Antibodies against NF‐κB P65 (AF5006), IκBα (AF5002), p‐NF‐κB P65 (AF2006), p‐IκBα (AF2002), TNF‐α (AF7014), IL‐6 (DF6087), IL‐1β (AF5103), MMP‐13 (AF5355), β‐tubulin (AF7011), β‐actin (AF7018), and Arg‐1 (DF6657) were purchased from Affinity Biosciences (Jiangsu, China). Collagen II (WL03082) was purchased from WANLEIBIO (Shenyang, China). PE‐Cy7 anti‐mouse CD86 antibody (FMN086‐01‐025) was purchased from 4A Blotech Co., Ltd. (Beijing, China). PE anti‐mouse CD206 (141705) antibody was purchased from BioLegend Inc. (California, USA). LPS, horseradish peroxidase (HRP)‐conjugated goat anti‐mouse IgG, and HRP‐conjugated goat anti‐rabbit IgG were purchased from Biosharp Biotechnology (Anhui, China). Recombinant mouse IL‐4 was purchased from Aladdin Biochemical Technology Co., Ltd. (Shanghai, China). All other reagents and chemicals were of analytical grade.

### Preparation of BR/JPH203 Nanoparticles (BRJ) and Opsonized Nanoparticles (IgG/BRJ)

BRJ and IgG/BRJ were prepared using a modified nanoprecipitation method. Briefly, BR and JPH203 (J) were mixed with *N*,*N*‐dimethylformamide (DMF) in a predefined molar ratio. The mixture solution was continuously stirred for 24 h in a dark condition at 25 °C. After 24 h, the mixture was diluted with double‐distilled water (H_2_O) in a 1:9 (v/v) ratio using a magnetic stirrer (250 rpm) under N_2_ protection. The mixture was dialyzed (MWCO: 3000 Da) against double‐distilled water for 24 h to remove the organic solution and produce BRJ. Next, IgG was diluted with the stirred BRJ solution (150 rpm) with different mass ratios of BR to IgG (1:0.05, 1:0.1, 1:0.2, 1:0.4, 1:0.5, and 1:1) to conjugate and produce IgG/BRJ. C6 was used to track the cellular uptake of IgG/BRJ. C6@BRJ and IgG/C6@BRJ were prepared similarly, except that an appropriate amount of C6 was added to the DMF solution.

### Molecular Docking Test

BR and JPH203 were subjected to molecular docking within the active sites of Mus musculus IgG (PDB code: 1IGT) using AutodockVina 1.2.2 software. Prior to the docking process, the 3D coordinates of IgG were acquired from the Protein Data Bank (PDB) website (http://www.rcsb.org/pdb/home/home.do). The molecular structures of BR and JPH203 were obtained from PubChem Compound (https://pubchem.ncbi.nlm.nih.gov/). For the docking analysis, both protein and molecular files were converted into the PDBQT format, excluding all water molecules, and polar hydrogen atoms were incorporated.

### Characterization of BRJ and IgG/BRJ

The size distribution, particle size, and zeta potential of BRJ and IgG/BRJ in the absence or presence of 0.3% H_2_O_2_ were determined using a NanoZetasizer (Zetasizer Nano, Malvern Panalytical, UK). The morphology of BRJ and IgG/BRJ in the absence or presence of 0.3% H_2_O_2_ was observed using TEM (JEM‐1200EX, JEOL, Japan) or SEM (SU8220, Hitachi, Japan). The stability of BRJ and IgG/BRJ was monitored by measuring the changes in the particle size and PDI of BRJ and IgG/BRJ nanoparticles dispersed in PBS (pH 7.4) at 25 °C for 1 week.

SDS‐PAGE was used to confirm the presence of IgG bound to the nanoparticle surface. Briefly, IgG, IgG/BRJ, and BRJ were subjected to electrophoresis in an 8% SDS‐PAGE gel, and the gel was stained with Coomassie Brilliant Blue, according to the manufacturer's instructions. The appearance of the gels was recorded. To determine the binding ratio of IgG to IgG/BRJ, the IgG/BRJ solution was centrifuged (18,000 rpm, 4 °C) for 1 h, and the supernatant was collected. The IgG concentration was detected using the BCA protein assay kit. The binding rate was calculated by subtracting the amount of free IgG.

The in vitro release curve of JPH203 from nanoparticles was determined using a modified dialysis method.^[^ ^33^ ^]^ Briefly, JPH203, BRJ, and IgG/BRJ were placed in a dialysis bag (MWCO: 3000 Da) and incubated in 25 mL of PBS (pH 7.4) containing 0.5% Tween 80 with or without 10 mM H_2_O_2_. The dialysis bag was placed in an oscillator (88A, Nuoji, China) and then shaken at 100 rpm (37 °C). Samples (10 mL) were collected at 10 min and 0.5, 1, 2, 4, 6, 8, 12, 24, 36, 48, 72, 96, and 120 h for concentration determination and were supplemented with the same volume of fresh medium. The concentration of JPH203 in the samples was determined by high‐performance liquid chromatography (Agilent, USA).^[^ ^26a^ ^]^

The interaction between BR and JPH203 was investigated using an UV–vis (UV6100S, MAPADA, China) and FTIR (iS10, Nicolet, USA).

### Cell Lines

Murine macrophage‐like cells (RAW 264.7) were obtained from ProCell Life Science and Technology Co., Ltd. (Hubei, China). Rat Cartilage Cells (RCC) were obtained from iCell Bioscience Inc. (Shanghai, China). RAW 264.7 was cultured in DMEM containing 10% fetal bovine serum (FBS) at 37 °C in a 5% CO_2_ condition. 1 µg/mL LPS was added to the medium of RAW 264.7 and incubated at 37 °C in a 5% CO_2_ atmosphere for 24 h to induce pro‐inflammatory M1 macrophages. RAW 264.7 cultured without LPS was represented as M0. RCC was cultured in DMEM/F12 containing 10% FBS, 100 units/mL streptomycin, and 100 units/mL penicillin at 37 °C in a 5% CO_2_ condition.

To compare the protection and therapeutic effect of IgG/BRJ and other formulations on RCC, RAW 264.7 conditioned media treated with normal media (M0M), LPS (M1M), LPS + BR (M1M‐1), LPS + JPH203 (M1M‐2), LPS + BR + J (M1M‐3), LPS + BRJ (M1M‐4), and LPS + IgG/BRJ (M1M‐5) for 48 h were collected. RAW 264.7 conditioned media were then incubated with RCC for 24 h for use in subsequent experiments. The untreated RCC group was used as the control group.

### QRT‐PCR Assay

The mRNA levels were measured by qRT‐PCR. Briefly, M0 macrophages were seeded in six‐well plates and incubated for 24 h until they reached 70–80% confluence. After treatment with LPS (1 µg mL^−1^) for 12 h, the cells were treated with different molar concentrations of BR (5, 10, and 20 µm), or JPH203 (5, 10, and 20 µm), or the mixture of BR and JPH203 in different ratios (1:0.5, 1:1, and 1:2; BR = 20 µm) for another 24 h. Subsequently, RNAiso plus (Takara, Japan) was used to extract total RNA. Total RNA was reverse transcribed using the reverse transcriptase kit (TOROIVD, China), according to the manufacturer's instructions. The SYBR Green qPCR master mix (TOROIVD, China) was used for qRT‐PCR, and Cq values were measured using a real‐time PCR instrument (Bio‐Rad, Hercules, CA, USA). The expression difference was calculated by the 2^−△△CT^ method. HPRT was used to normalize the expression of the target genes. Primers used for qRT‐PCR are as follows: HPRT, 5′‐GCGTCGTGATTAGCGATGATGAAC‐3′ (forward), 5′‐CCTCCCATCTCCTTCATGACATCT‐3′ (reverse); iNOS, 5′‐TGCTATTCCCAGCCCAAC‐3′ (forward), 5′‐GGTGAAGGGTGTCGTGAAA‐3′ (reverse); TNF‐α, 5′‐CGCTGAGGTCAATCTGC‐3′ (forward), 5′‐GGCTGGGTAGAGAATGGA‐3′ (reverse); IL‐1β, 5′‐TGCCACCTTTTGACAGTGATG‐3′ (forward), 5′‐ATGTGCTGCTGCGAGATTTG‐3′ (reverse); IL‐10, 5′‐GGCGCTGTCATCGATTTCTC‐3′ (forward), 5′‐GCCTTGTAGACACCTTGGTCTT‐3′ (reverse); Arg‐1, 5′‐CCACAGTCTGGCAGTTGGAAG‐3′ (forward), 5′‐GGTTGTCAGGGGAGTGTTGATG‐3′ (reverse).

### In Vitro Cellular Uptake Assay

C6 was selected as the probe to indicate the uptake of nanoparticles by cells. M0 or M1 macrophages were seeded in 12‐well plates and cultured for 24 h until they reached 60–70% confluence. The cells were then incubated with free C6, C6@BRJ, or IgG/C6@BRJ (C6 dose: 1.5 µg mL^−1^) for 1, 2, and 4 h at 37 °C, respectively. After washing with PBS three times, the cells were fixed with 4% paraformaldehyde. The cells were washed again with PBS three times, and visualized under a fluorescence microscope (Nikon, Japan), and the mean fluorescence intensity of the images were quantitatively analyzed using ImageJ software.

### In vitro cytocompatibility

The safety of BRJ and IgG/BRJ to cells was assessed using the MTT assay. Briefly, M0 macrophages, M1 macrophages, and RCC cells were inoculated into 96‐well plates at a density of 5 × 10^3^ cells per well for 24 h. The cells were then incubated with different concentrations of BRJ or IgG/BRJ for an additional 24 or 48 h. The cells were then incubated with MTT solution (5 mg/mL) at 37 °C for 4 h, and 100 µL of DMSO was added into the well to dissolve the generated formazan. Finally, the absorbance at 490 nm was measured using a microplate analyzer (Infinite M200 Pro, Switzerland), and cell viability was calculated.

### Intracellular ROS Assay

Intracellular ROS levels were detected using a ROS assay kit. In brief, M0 macrophages were seeded in 12‐well plates and cultured for 24 h until 60–70% confluence. The cells were treated with LPS for 12 h to induce M1 polarization. Subsequently, the cells were incubated with BR, JPH203, BR+J, BRJ, or IgG/BRJ (BR: JPH203 = 1:0.5, BR = 20 µm) for additional 24 h. Untreated cells served as the control. After washing three times with PBS, the cells were incubated with ROS probe for 30 min at 37 °C. The cells were washed again three times with PBS then observed and photographed under a fluorescence microscope (Nikon, Japan). The mean fluorescence intensity of the images was quantified using the ImageJ software. For RCC, cells were seeded in 12‐well plates and cultured for 24 h to 60–70% confluence. The cells were then incubated with conditioned media of M0M, M1M, M1M‐1, M1M‐2, M1M‐3, M1M‐4, and M1M‐5, as section cell lines described, for 24 h.

### WB Assay

The WB technique was used to analyze specific protein expression to determine the anti‐inflammatory effect and chondrocyte protection of IgG/BRJ. In brief, RAW 264.7 or RCC were seeded in six‐well plates and incubated for 24 h until 70–80% confluence. The cells were treated using the same procedure as section Intracellular ROS assay described. Untreated cells were used as the control. Cell lysates were prepared in radioimmunoprecipitation (RIPA) buffer containing 1% protease and 1% phosphatase inhibitors. The protein concentration was detected using a BCA kit. Proteins were separated using SDS‐PAGE and transferred onto a polyvinylidene difluoride (PVDF) membrane. The PVDF membrane was then blocked with 0.1% Tween 20 Tris buffer (TBST) containing 5% BSA at 25 °C for 1 h, followed by overnight incubation with primary antibodies at 4 °C. After the membranes were washed three times for 5 min each with TBST, they were incubated with HRP‐conjugated secondary antibodies at room temperature for 1 h. After washing three times for 5 min each with TBST, the membranes were soaked in ECL substrate and visualized using an exposure machine (Bio‐Rad, USA). The following steps were performed as previously described. Protein band quantification was performed using the AlphaView software.

### IF Assay

IF was used to assess the effect of IgG/BRJ on the regulation of macrophage polarization and the upregulation of RCC anabolism. The RAW 264.7 or RCC were seeded in 12‐well plates with 18‐mm round coverslips (Biosharp, China) and incubated for 24 h until they reached 60–70% confluence. The cells were treated using the same procedure as section Intracellular ROS assay described. Untreated cells were used as the control. After washing three times with PBS, the cells were fixed with 4% paraformaldehyde for 20 min at 37 °C and permeabilized with 0.1% Triton‐X 100 (Biosharp, China) at 25 °C for 10 min. The cells were then blocked with 10% BSA at 37 °C for 1 h and incubated with primary antibodies at 4 °C for 16 h. After washing three times with PBST, the cells were incubated with AlexaFluor488 goat anti‐rabbit IgG (Biosharp, China) for 1 h and then washed with PBST three times. An anti‐fade mounting medium containing DAPI was used to seal the coverslips. Finally, images were taken using a fluorescence microscope (Nikon, Tokyo, Japan), and the mean fluorescence intensity of the images was quantified using ImageJ software.

### Flow Cytometry

Flow cytometry was used to assess the effects of IgG/BRJ on macrophage polarization. The RAW 264.7 was seeded in six‐well plates and cultured for 24 h until they reached 60–70% confluence. The cells were treated using the same procedure as section Intracellular ROS assay described. After washing three times with PBS, 500 µL of PBS containing 2% BSA was added to flush the cells, and 1 × 10^5^ cells were counted. The cells were resuspended in 500 µL of PBS containing 2% BSA and incubated antibody at 25 °C for 30 min. After washing three times, the cells were resuspended in 300 µL of PBS containing 2% BSA. The cells were detected by flow cytometry (Beckman, USA) and analyzed using FlowJo software.

### Metabonomics Analysis

M0 macrophages were initially cultured in 100 mm culture dishes for 24 h until they reached 70–80% confluence. Subsequently, the cells were treated with LPS (2 µg mL^−1^) for 12 h to induce M1 polarization or with IL‐4 (40 ng mL^−1^) for 12 h to induce M2 polarization. Following this, the M1 macrophages were further incubated with IgG/BRJ (BR: JPH203 = 1:0.5, BR = 40 µm) for an additional 24 h. Untreated cells were used as the control group. After digestion with pancreatic enzymes, cells were rinsed with PBS for recapitulation and accurate cell counting. An equivalent number of cells (1 × 10^7^) was packed into new 1.5 mL centrifuge tubes. The cells were then centrifuged at 800 × g for 10 min. After discarding the supernatant, any residual liquid present was carefully removed. The cells were subjected to quick freezing using liquid nitrogen, and stored at −80 °C. The samples were analyzed by Shanghai Majorbio Bio‐Pharm Technology Co., Ltd. Data were analyzed using the Majorbio Cloud Platform (www.majorbio.com).

### OA Model and Macroscopic Evaluation

Male Sprague–Dawley (SD) rats (240‐280 g) were purchased from the Laboratory Animal Center of Wenzhou Medical University. All animal studies were conducted in accordance with the Guiding Principles of Animal Experiments at Wenzhou Medical University and approved by the Animal Ethics Committee of Wenzhou Medical University (xmsq2023‐0326). An ACLT of the right knee of rats was performed to establish the OA model. Sham‐operated rats were used as controls, as previously described.^[^ ^44^ ^]^ The model was validated using a front‐drawer test. At 1 month post‐operative, ACLT rats were randomly divided into five groups (n = 7) for intra‐articular injection of different preparations: 1) saline, 2) BR+J, 3) BRJ, 4) IgG/BRJ, and 5) HA, and sham‐operated rats were used as healthy controls. Ratio of BR and JPH203 was 1:0.5 (100 and 50 µm concentrations of BR and JPH203, respectively) in each preparation. The concentration of HA was and 5 mg mL^−1^.^[^ ^45^ ^]^ The rats in each group received intra‐articular administration of 100 µL of the preparation on days 35, 40, 45, 50, 55, and 60. All rats were euthanized on day 65, and the joints were removed and collected for micro‐CT (SKYSCAN 1276, BRUKER, Germany) imaging. Macroscopic scores were determined using a previously reported method.^[^ ^46^ ^]^

### Histological Analysis and IHC

Knee joints and synovium were collected from euthanized rats and fixed in 4% paraformaldehyde for 48 h. After rinsing overnight, the knee joint was immersed in EDTA decalcification solution for 1 month, and the synovium was dehydrated and embedded. The knee joint and synovium were sectioned into 5‐µm paraffin slices, and the synovium and cartilage slices were routinely dewaxed and stained with H&E. Cartilage slices were routinely dewaxed and stained with modified safranin O and toluidine blue to evaluate the GAGs content and cartilage morphology. Histomorphology was visualized and recorded using a microscope (Nikon, Tokyo, Japan). In addition, the OARSI score was assessed according to reported guidelines.^[^ ^47^ ^]^

For IHC, 5‐µm paraffin slices were dewaxed routinely and incubated with 3% H_2_O_2_ at 25 °C for 15 min to remove endogenous peroxidase. The sections were then incubated with 0.25% EDTA trypsin at 37 °C for 20 min for antigen repair. The slices were blocked with 10% goat serum at 37 °C for 1 h and then incubated for 16 h with the primary antibodies in a wet box at 4 °C. After incubation, the wet box was rewarmed for 30 min, and the slides were washed twice with PBS for 5 min each. Subsequently, the slices were incubated with the secondary antibodies for 20 min at 37 °C and developed DAB stain with an IHC kit. Secondary antibody incubation and DAB color development were performed using an IHC kit. Finally, images were captured with a microscope (Nikon, Japan), and the proportion of positive areas was calculated using Image‐Pro Plus software.

### In Vivo Retention Assessment

Nine male SD rats (240‐280 g) were used to establish the OA model using the ACLT method in the right knee joints and sham‐operated in the left knee joints. After 2 months, the ACLT rats were randomly divided into the following three groups: 1) Free DiR, 2) DiR@BRJ, and 3) IgG/DiR@BRJ (n = 3 in each group) to detect the retention ability of these formulations in healthy and OA knee joints. Each rat received 5 µg of DiR. The rat knee joints were imaged using the in vivo imaging system (Caliper Life Sciences, USA) at 12, 24, 48, 72, 120, and 168 h after injection.

### Statistical Analysis

All statistical analyses were performed using GraphPad Prism 8.0 software (La Jolla, CA, USA). Statistical analysis was performed using the Student's *t*‐test or one‐way ANOVA. Data are presented as mean ± standard deviation (SD) (a *P* < 0.05 indicates statistical significance).

## Conflict of Interest

The authors declare no conflict of interest.

## Author Contributions

H.H., S.Z., and J.W. contributed equally to this work. H.H. designed methodology; developed software; performed validation; wrote the original draft. S.Z. designed methodology; performed validation and investigation. J. W. designed methodology; developed software; and performed investigation. X.L. designed methodology. S.L., P.M., X.Z., and A.C. designed methodology. Z.H., Y.C., and L.S. performed validation. L.W. wrote the original draft. H.S. performed validation. Q.Y. wrote the original draft. R.C. performed funding acquisition; performed supervision and project administration. Y.Z. performed project administration and supervision. L.K. performed funding acquisition, supervision, and project administration, and wrote the original draft.

## Supporting information

Supporting Information

## Data Availability

The data that support the findings of this study are available from the corresponding author upon reasonable request.
