# Supplementary material for: Opsonization Inveigles Macrophages Engulfing Carrier‐Free Bilirubin/JPH203 Nanoparticles to Suppress Inflammation for Osteoarthritis Therapy
Source: Adv Sci (Weinh). 2024 Apr 9;11(22):2400713. doi: 10.1002/advs.202400713 (PMC11165524; doi:10.1002/advs.202400713)
Supplement: Supplementary file 1 — Supporting Information [file ADVS-11-2400713-s001.docx]

Supplement Materials

**Opsonization Inveigles Macrophages Engulfing Carrier-free Bilirubin/JPH203 Nanoparticles to Suppress Inflammation for Osteoarthritis Therapy**

*Huirong Huang, Shimin Zheng, Jianing Wu, Xindan Liang, Shengjie Li, Pengfei Mao, Zhinan He, Yahui Chen, Lining Sun, Xinyu Zhao, Aimin Cai, Luhui Wang, Huixiang Sheng, Qing Yao, Ruijie Chen*,* *Ying-Zheng Zhao*, Longfa Kou**

H. Huang, S. Zheng, J. Wu, X. Liang, S. Li, P. Mao, Z. He, Y. Chen, L. Sun, X. Zhao, A. Cai, H. Sheng, Q. Yao, R. Chen, L. Kou

Wenzhou Municipal Key Laboratory of Pediatric Pharmacy

Department of Pharmacy

The Second Affiliated Hospital and Yuying Children's Hospital of Wenzhou Medical University

Wenzhou 325027, China

E-mail: koulongfa@wmu.edu.cn; crj@wzhealth.com

H. Huang, S. Zheng, J. Wu, X. Liang, S. Li, P. Mao, Z. He, Y. Chen, L., X. Zhao, A. Cai, R. Chen, L. Kou

Key Laboratory of Structural Malformations in Children of Zhejiang Province

Wenzhou 325027, China

H. Huang, X. Liang, S. Li, Z. He, Y. Chen, L. Sun, Q. Yao, Y. Zhao

School of Pharmaceutical Sciences

Wenzhou Medical University

Wenzhou 325035, China

E-mail: zhaoyz@wmu.edu.cn

L. Wang

Department of Ultrasonography

The First Affiliated Hospital of Wenzhou Medical University

Wenzhou 325015, China


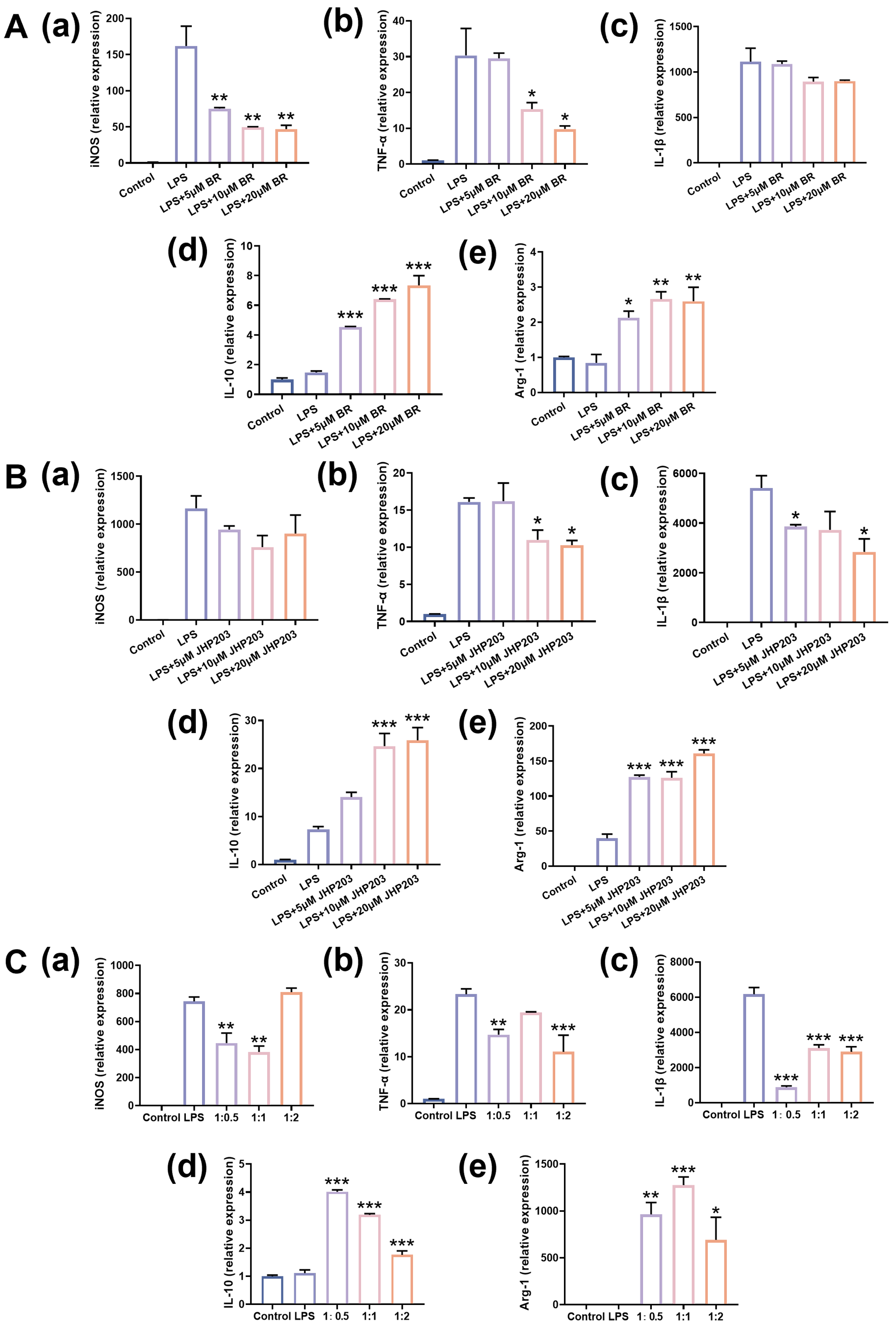


**Figure S1.** (A) BR onset concentration. The mRNA level of (a) iNOS, (b) TNF-α, (c) IL-1β, (d) IL-10 and (e) Arg-1 in LPS activated RAW 264.7 with different molar concentrations of BR. (B) JPH203 onset concentration. The mRNA level of (a) iNOS, (b) TNF-α, (c) IL-1β, (d) IL-10 and (e) Arg-1 in LPS activated RAW 264.7 with different molar concentrations of JPH203. (C) Combination ratio of BR and JPH203. The mRNA level of (a) iNOS, (b) TNF-α, (c) IL-1β, (d) IL-10 and (e) Arg-1 in LPS activated RAW 264.7 with different molar ratios of BR to JPH203. Data are expressed as mean ± SD (n = 2). * *P* < 0.05, ** *P* < 0.01, *** *P* < 0.001.


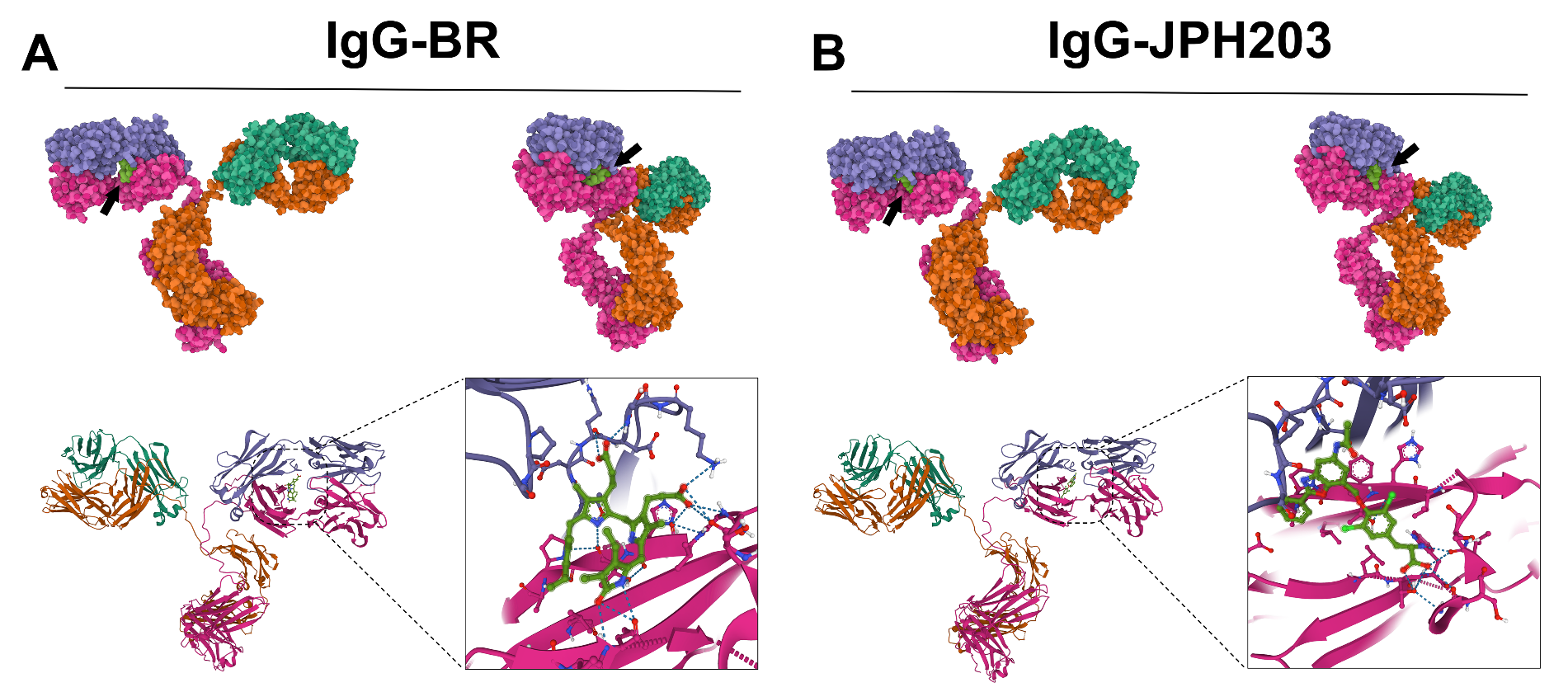


**Figure S2.** Assessment the affinity of BR and JPH203 to IgG by molecular docking analysis. (A) Binding mode of IgG and BR (green). Hydrogen bonding (blue dotted line) at THR 156; TRP 157; GLY 164; ASP 165; GLN 166; LEU 166; SER 167; VAL 171; SER 168; LYS 169; THR 173; PHE 174. (B) Binding mode of IgG and JPH203 (green). Hydrogen bonding (blue dotted line) at THR 156; GLY 164; LEU 166.


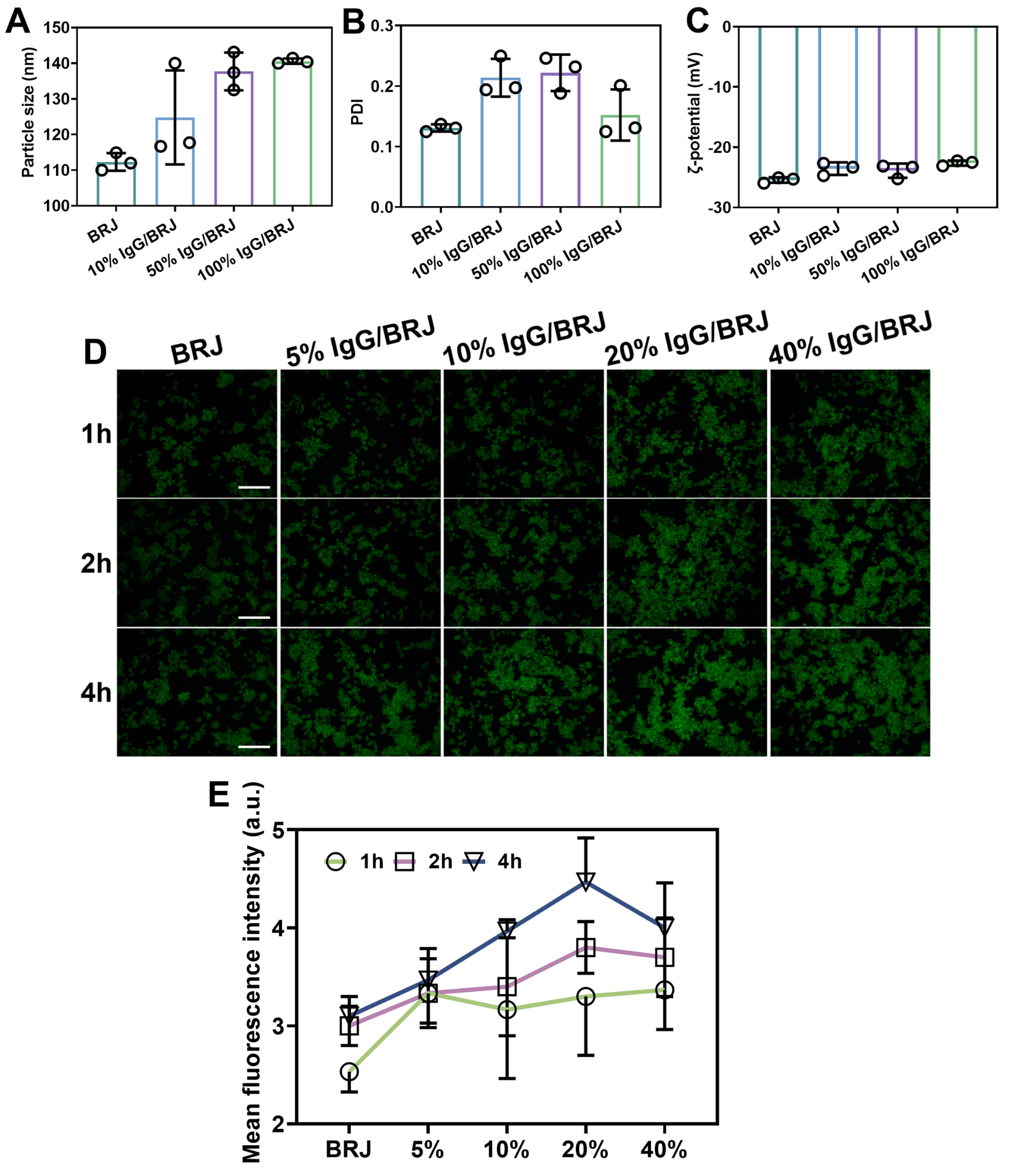


**Figure S3.** Optimum adsorption IgG ratio search. (A) The particle size of 10% IgG/BRJ, 50% IgG/BRJ and 100% IgG/BRJ nanoparticles. (B) The PDI of 10% IgG/BRJ, 50% IgG/BRJ and 100% IgG/BRJ nanoparticles. (C) The ζ-potential of 10% IgG/BRJ, 50% IgG/BRJ and 100% IgG/BRJ. (D) Fluorescence images of free C6 and BRJ, 5% IgG/BRJ, 10% IgG/BRJ, 20% IgG/BRJ, 40% IgG/BRJ nanoparticles (C6: green) ingested by M1 macrophage after 1 h, 2 h and 4 h respectively. Scale bar = 100 μm. (E) Quantitative analysis of (D) by using image J software. Data are expressed as mean ± SD (n = 3).


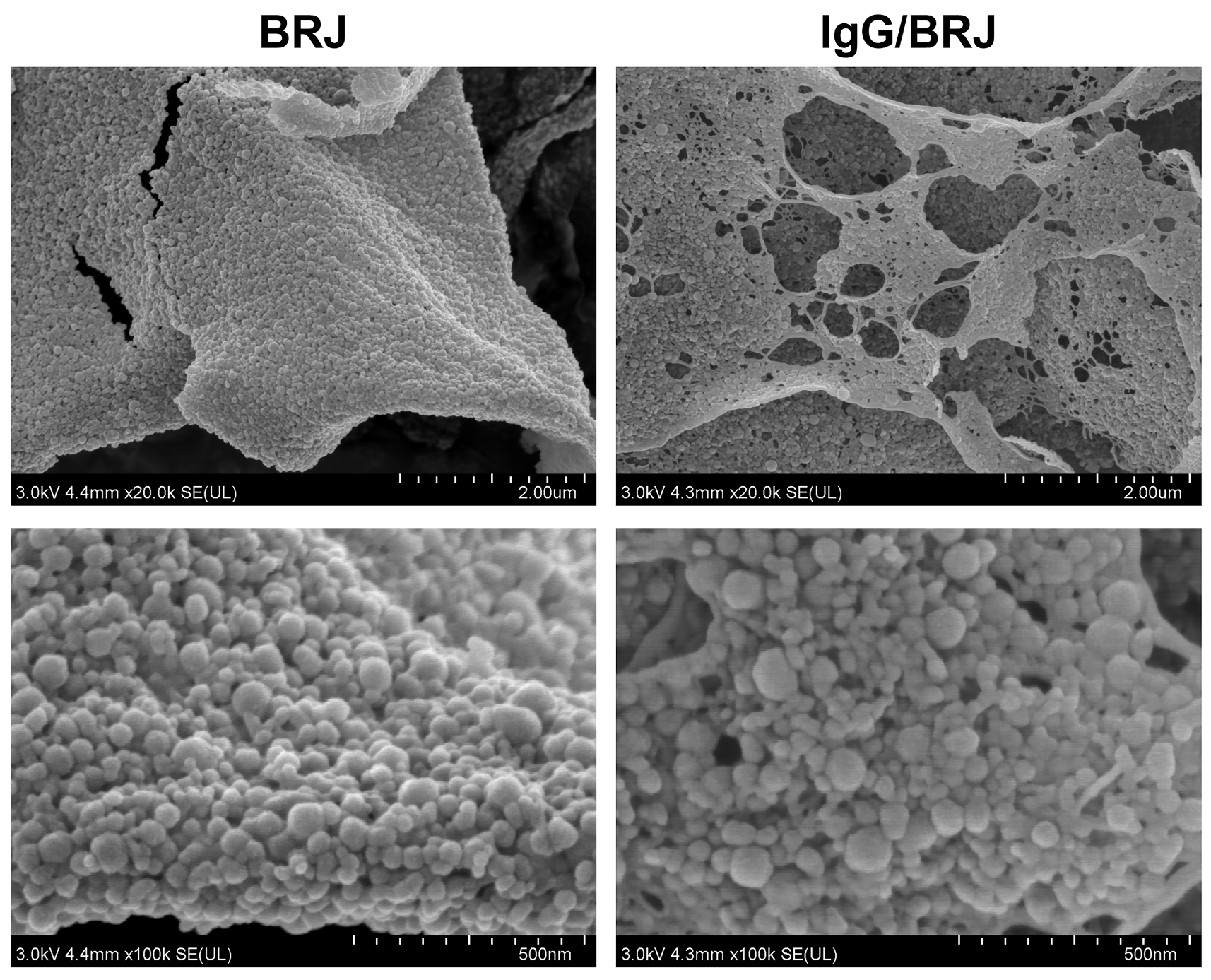


**Figure S4.** SEM images of BRJ and IgG/BRJ.


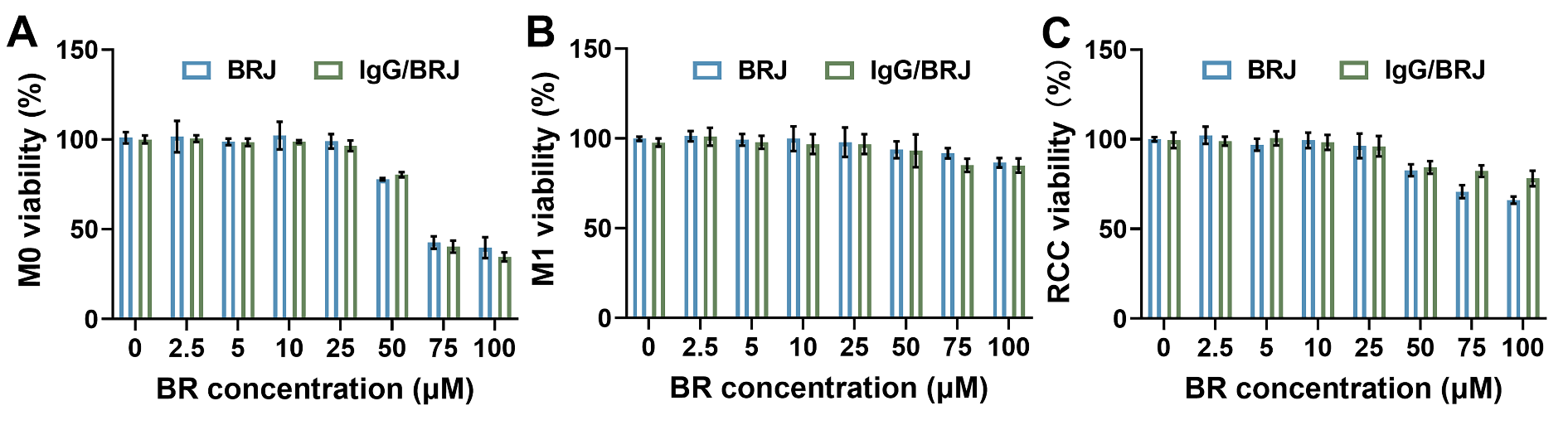


**Figure S5.** Cell safety of BRJ and IgG/BRJ. (A) M0, (B) M1, and (C) RCC after treatment with BRJ or IgG/BRJ for 48 h. Data are expressed as mean ± SD (n = 3).


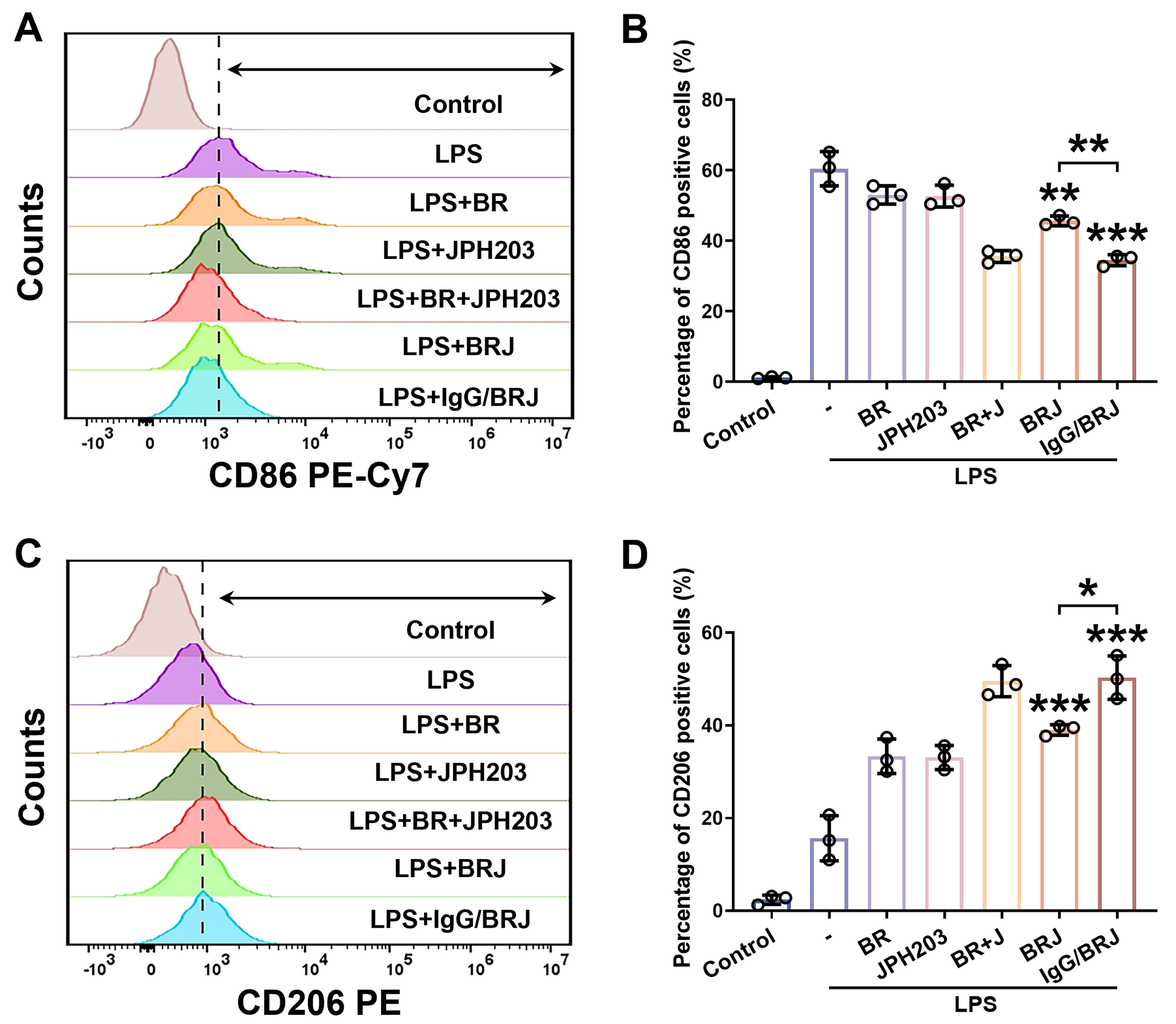


**Figure S6.** IgG/BRJ promote macrophage polarization from M1 to M2. Flow cytometry was used to quantify the expression of (A, B) CD86 (an M1 marker) and (C, D) CD206 (an M2 macrophage marker). Data in B and D are expressed as mean ± SD (n = 3), * *P* < 0.05, ** *P* < 0.01, *** *P* < 0.001, compared to LPS group or as indicated.


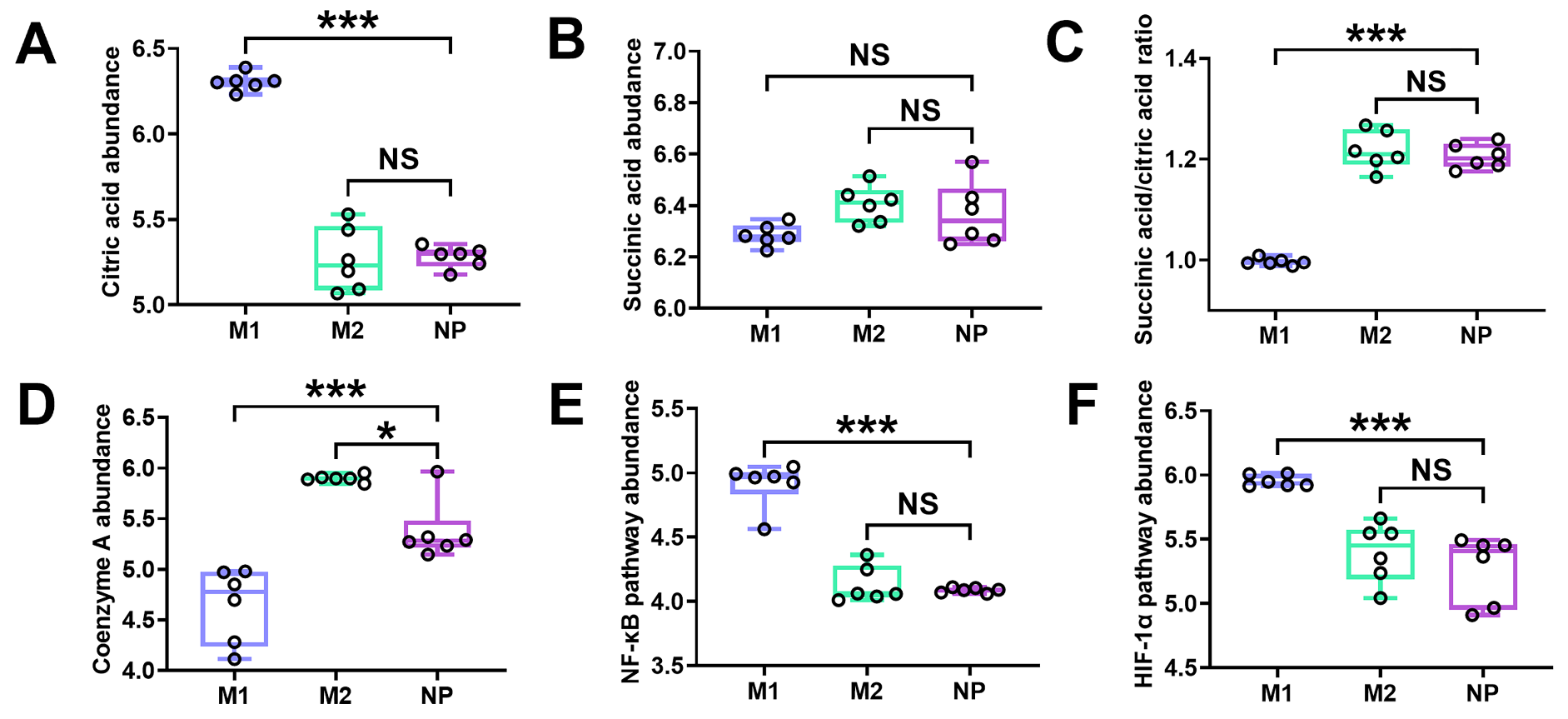


**Figure S7**. In vitro metabolic regulation effects of IgG/BRJ in RAW 264.7. (A-F) Quantification analysis the abundance of citric acid, succinic acid, the ratio of succinic acid to citric acid, coenzyme A, NF-κB pathway-related metabolites, HIF-1α pathway-related metabolites, respectively. Data are expressed as mean ± SD (n = 6). NS *P* > 0.05, * *P* < 0.05, *** *P* < 0.001, compared as indicated.


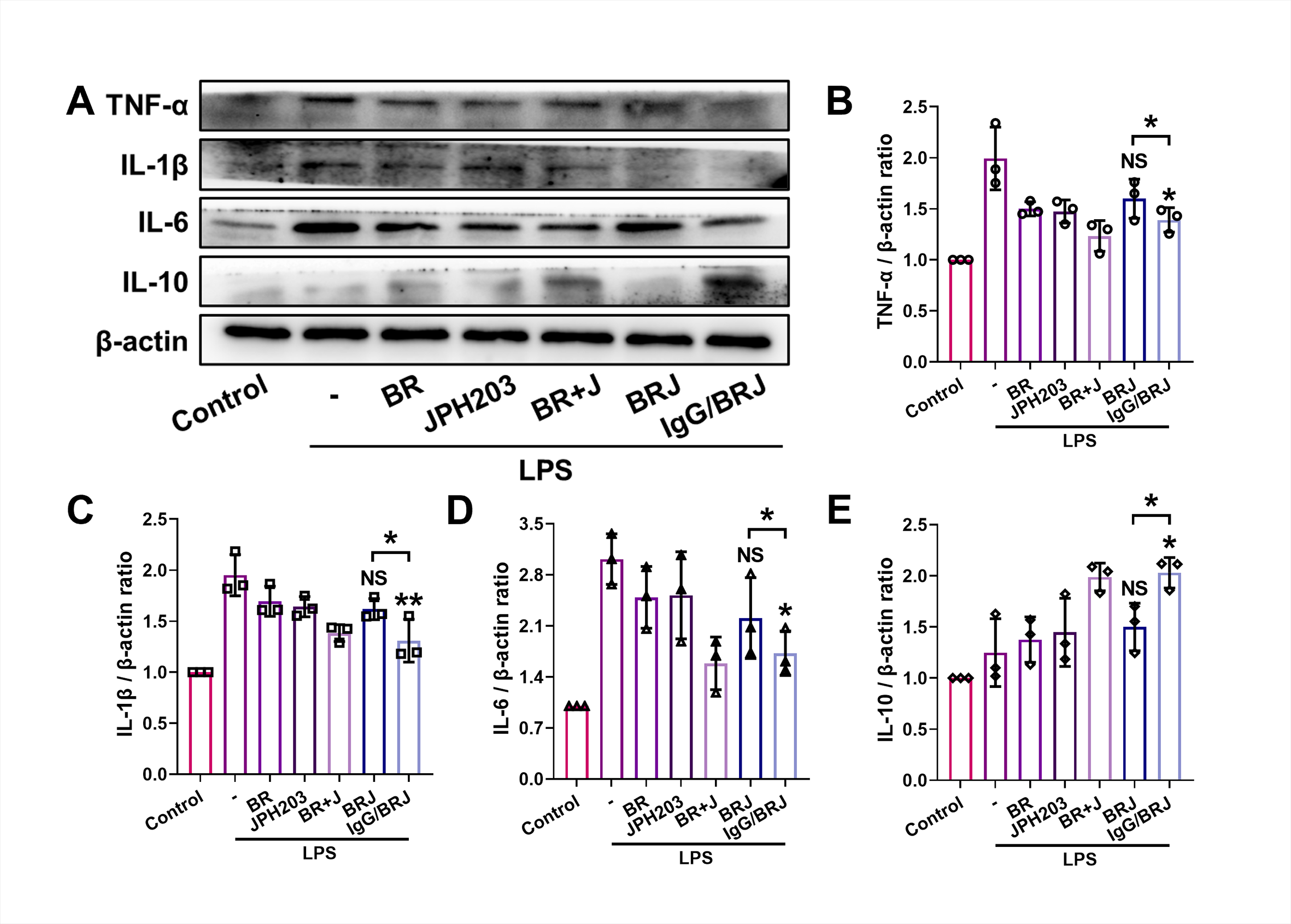


**Figure S8**. In vitro anti-inflammatory effects of IgG/BRJ in RAW 264.7. (A) The expression of TNF-α, IL-1β, IL-6, IL-10 in RAW 264.7 after LPS stimulation and treatment with different formulations. (B-E) Quantification analysis of (A). Data are expressed as mean ± SD (n = 3). NS *P* > 0.05, * *P* < 0.05, ** *P* < 0.01, compared to the LPS groups or as indicated.

**
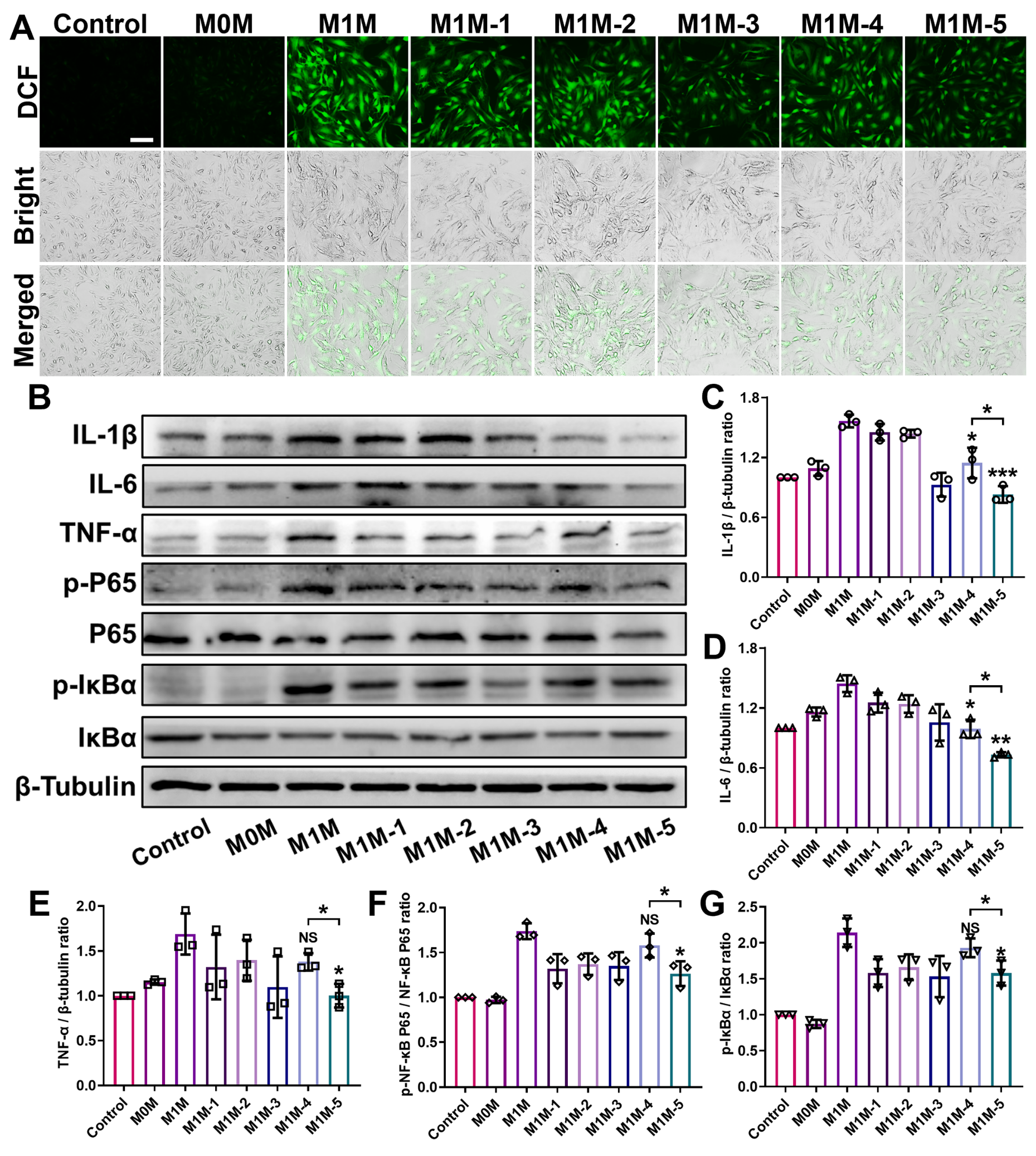
**

**Figure S9**. IgG/BRJ reduces ROS level and inflammatory level in RCC cells. (A) ROS level in RCC cells after different treatments were detected by DCFH-DA probe. Scale bar = 100 μm. (B) The expression of IL-1β, IL-6, TNF-α, p-P65, P65, p-IκBα and IκBα in RCC after treatment with different conditional medium culture. (C-G) Quantification analysis of (B). Data are expressed as mean ± SD (n = 3). NS *P* > 0.05, * *P* < 0.05, *** *P* < 0.001, compared to M1M group or as indicated. Control: normal RCC; M0M: RCC cultured in M0 conditioned medium culture; M1M: RCC cultured without treatment in M1 conditioned medium; M1M-1: RCC cultured in BR-treated M1 conditioned medium; M1M-2: RCC cultured in JPH203-treated M1 conditioned medium; M1M-3: RCC cultured in BR+J-treated M1 conditioned medium; M1M-4: RCC cultured in BRJ-treated M1 conditioned medium; M1M-5: RCC cultured in IgG/BRJ-treated M1 conditioned medium.


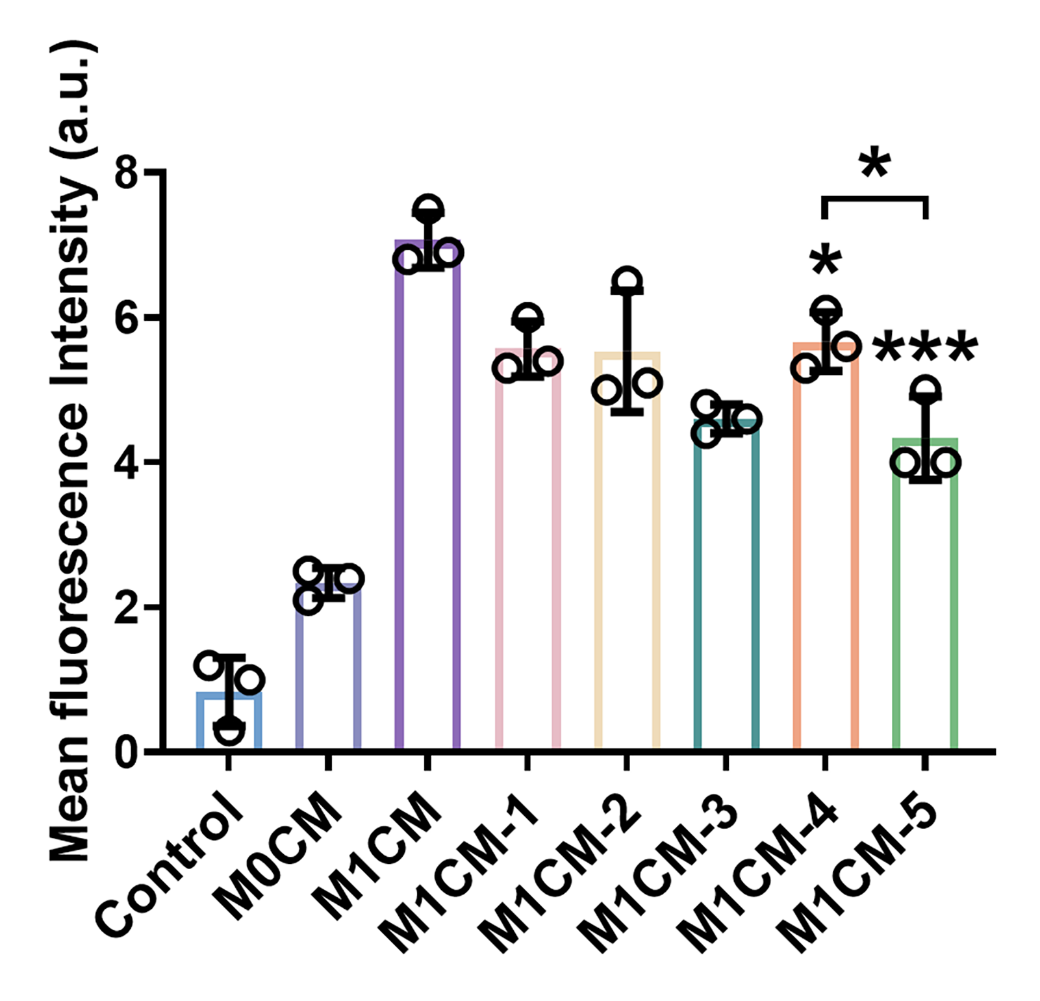


**Figure S10.** The fluorescence intensity of DCF was quantitatively analyzed by ImageJ software. Data are expressed as mean ± SD (n = 3). * *P* < 0.05, *** *P* < 0.001, compared to M1M group or as indicated. Control: normal RCC; M0M: RCC cultured in M0 conditioned medium culture; M1M: RCC cultured without treatment in M1 conditioned medium; M1M-1: RCC cultured in BR-treated M1 conditioned medium; M1M-2: RCC cultured in JPH203-treated M1 conditioned medium; M1M-3: RCC cultured in BR+J-treated M1 conditioned medium; M1M-4: RCC cultured in BRJ-treated M1 conditioned medium; M1M-5: RCC cultured in IgG/BRJ-treated M1 conditioned medium.


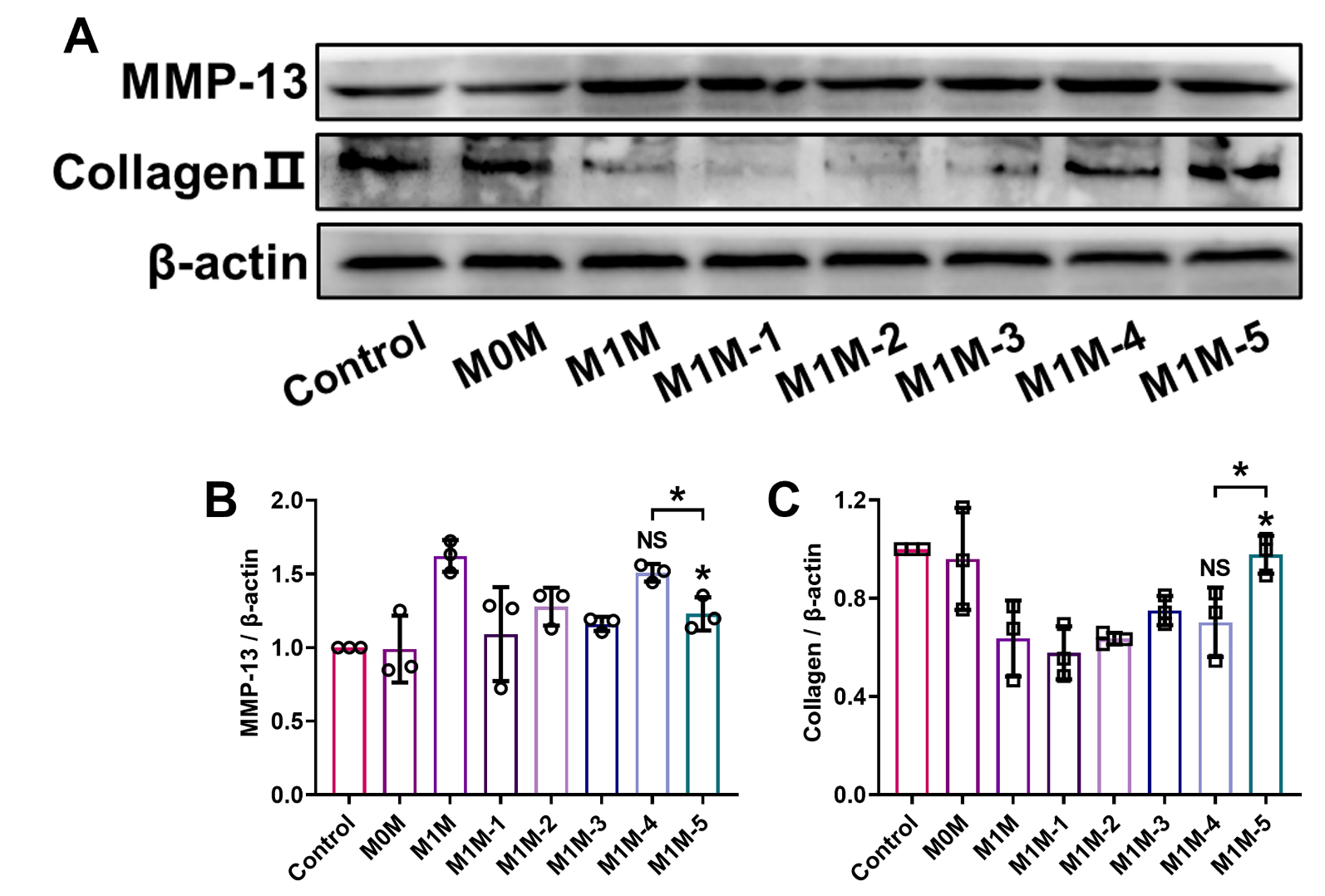


**Figure S11.** IgG/BRJ enhance anabolism level of RCC. (A) The expression of Collagen Ⅱ and MMP-13 in RCC after treatment with different conditional medium culture. (B, C) Quantitative analysis of (A). Data are expressed as mean ± SD (n = 3). NS *P* > 0.05, * *P* < 0.05, compared to M1M group or as indicated. Control: normal RCC; M0M: RCC cultured in M0 conditioned medium culture; M1M: RCC cultured without treatment in M1 conditioned medium; M1M-1: RCC cultured in BR-treated M1 conditioned medium; M1M-2: RCC cultured in JPH203-treated M1 conditioned medium; M1M-3: RCC cultured in BR+J-treated M1 conditioned medium; M1M-4: RCC cultured in BRJ-treated M1 conditioned medium; M1M-5: RCC cultured in IgG/BRJ-treated M1 conditioned medium.


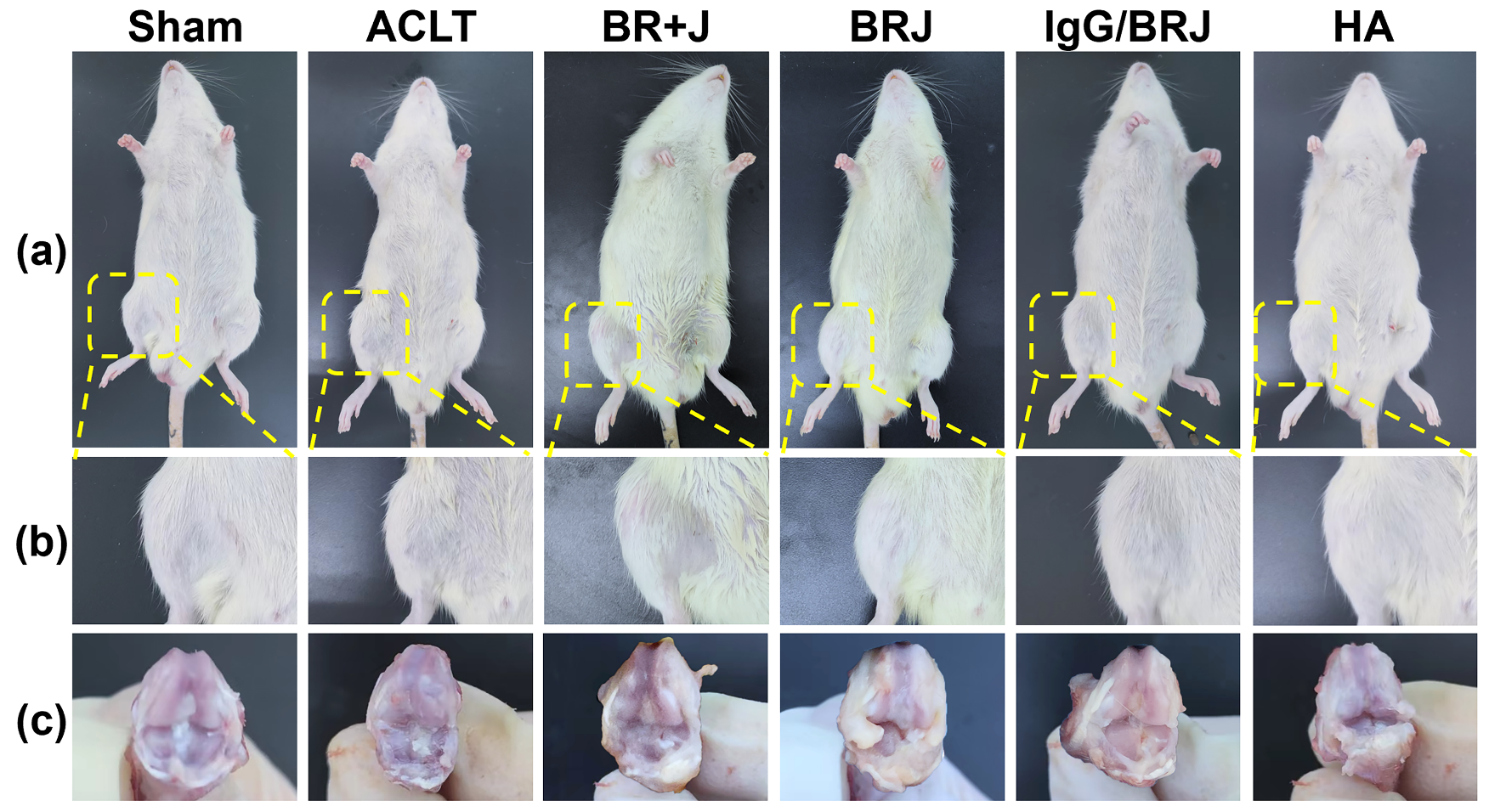


**Figure S12.** Macroscopic observation of rat after 65 days of intra-articular injection of saline, BR+J, BRJ, IgG/BRJ, and HA. (a) Overall image of the rat. (b) Enlarged image of the rat knee joint. (c) Cartilage close-up of rat cartilage after dissected.


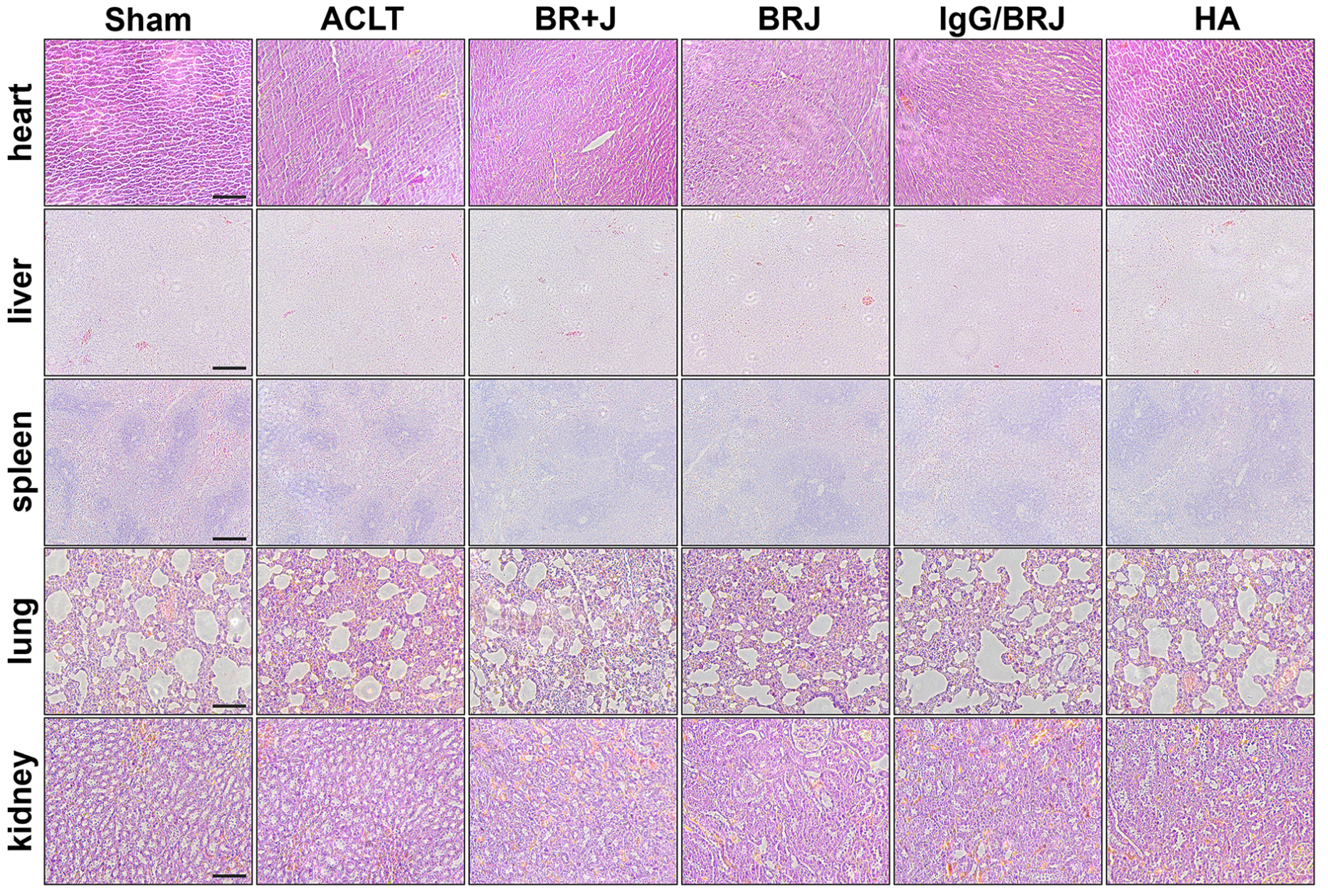


**Figure S13.** H&E staining of the major organs after various treatments. Scale bar = 100 μm.
